# Ethnicity and antenatal maternal anxiety: associations with the human milk lipidome in a multi-ethnic Asian cohort

**DOI:** 10.3389/fnut.2026.1908654

**Published:** 2026-08-20

**Authors:** Jayashree Selvalatchmanan, Shanshan Ji, Li Chen, Helen Chen, Mary F. F. Chong, Johan G. Eriksson, Yap-Seng Chong, Mei Chien Chua, Kok Hian Tan, David Bradley, Markus Wenk, Mary E. Wlodek, Shiao-Yng Chan, Wei Wei Pang, Federico Torta

**Affiliations:** 1 Singapore Lipidomics Incubator, Department of Biochemistry, Yong Loo Lin School of Medicine, National University of Singapore, Singapore, Singapore; 2 Agency for Science, Technology and Research (A*STAR) Institute for Human Development and Potential, Singapore, Singapore; 3 Department of Psychological Medicine, KK Women’s and Children’s Hospital, Singapore, Singapore; 4 Duke-NUS Medical School, Singapore, Singapore; 5 Saw Swee Hock School of Public Health, National University of Singapore and National University Health System, Singapore, Singapore; 6 Folkhälsan Research Center, Helsinki, Finland; 7 Department of General Practice and Primary Health Care, University of Helsinki, Helsinki, Finland; 8 Department of Obstetrics and Gynecology, Yong Loo Lin School of Medicine, National University of Singapore, Singapore, Singapore; 9 Department of Maternal Fetal Medicine, KK Women’s and Children’s Hospital, Singapore, Singapore; 10 Pacific Technology Marketing Pty. Ltd., Lucaston, TAS, Australia; 11 College of Health and Life Sciences, Hamad Bin Khalifa University, Doha, Qatar; 12 Department of Obstetrics and Gynecology, The University of Melbourne, Parkville, VIC, Australia; 13 School of Molecular Sciences, The University of Western Australia, Perth, WA, Australia; 14 Global Centre for Asian Women’s Health (GloW), Yong Loo Lin School of Medicine, National University of Singapore, Singapore, Singapore; 15 Signature Research Program in Cardiovascular and Metabolic Disorders, Duke-NUS Medical School, Singapore, Singapore; 16 Precision Medicine Translational Research Programme, Department of Biochemistry, Yong Loo Lin School of Medicine, National University of Singapore, Singapore, Singapore

**Keywords:** antenatal anxiety, ceramides, ethnicity, human milk, lipidomics, polyunsaturated fatty acids

## Abstract

**Introduction:**

Human milk (HM) lipid composition varies considerably among women, but factors associated with variation in complex lipids beyond fatty acid constituents are less well-described. We investigated maternal and infant factors associated with the intact human milk lipidome at 3 weeks and 3 months postpartum in a multi-ethnic Asian population.

**Methods:**

In this observational cohort study, 237 lipids across 12 lipid subclasses were quantified in HM samples collected at 3 weeks (*n* = 189) and 3 months (*n* = 104) postpartum via liquid chromatography-mass spectrometry and direct-infusion mass spectrometry. Principal component analysis captured major axes of lipid variation and time-specific regression models examined associations between predictors (including ethnicity, parity, pre-pregnancy BMI, tobacco smoke exposure, maternal mental health, breastfeeding exclusivity/predominance) and top principal components, with correction for multiple comparisons.

**Results:**

Ethnicity emerged as the strongest factor at both timepoints, explaining 5.6%–10.4% of the variance at 3 weeks and 2.6%–15.6% at 3 months. Chinese had higher polyunsaturated-fatty-acid (PUFA) lipids, especially very long-chain PUFA triacylglycerols (TGs) and n-3 PUFA phospholipids (PLs), than Indians and Malays. Indians had higher odd-chain lipids (saturated and monounsaturated) and specific PLs compared with Chinese. Together, Indians and Malays had a more saturated- and monounsaturated lipid composition, as well as long-chain sphingolipids compared with Chinese. Antenatal maternal anxiety was independently associated with an altered lipid profile - higher ceramides, monounsaturated lipids and lower PUFA TGs – at 3 weeks postpartum.

**Conclusion:**

Ethnic differences may partly reflect culturally distinct postpartum confinement diets and fatty acid desaturase (FADS1/FADS2) genotypic variants. Further investigation into culturally tailored nutritional guidance and perinatal psychological support as modifiable factors associated with the HM lipidome is warranted.

## Introduction

The Developmental Origins of Health and Disease (DOHaD) theory emphasizes the importance of early life exposures in shaping the long-term health trajectory of individuals (1, 2). Within this framework, understanding human milk (HM) composition is valuable as it reflects a multitude of maternal factors, and is an infant’s first food. HM composition is dynamic and known to vary considerably between mothers; it is shaped not only by genetics but also by maternal, environmental, and lactation factors, and is amenable to modification (3, 4). However, to inform guidelines, such as those pertaining to personalized nutritional and lactation advice for mothers and mothers-to-be, that may eventually affect downstream child outcomes, it is important to characterize the factors that are associated compositional differences in HM.

Although constituting only 2%–5% of mature milk, lipids are the predominant source of energy (∼50%) for the growing infant. HM lipids are key nutrients for healthy growth and development, and additionally act as bioactive signals in their own right. In HM, lipid species are mainly proportioned as 98%–99% triacylglycerols (TG), and 1%–2% phospholipids (PL) and sphingolipids (SP) (5, 6). The overwhelming presence of TGs, combined with methodological limitations, has constrained research on intact lipids; many studies typically quantify total TGs or their constituent fatty acids (7–9). Analyzing lipids only at the fatty acid level may overlook differences in biological function across the lipid subclasses arising from their differential structures, digestive routes, and bioactive functions (10–12). Moreover, beyond the nutritional role that lipids in HM are often thought to play, HM lipids may also reflect maternal metabolic and environmental influences (13, 14). It is therefore important to understand how maternal factors are reflected in variation in the intact HM lipidome.

While the variability of fatty acids and their associations with various maternal factors have been studied by many groups globally (4, 15), far less is known about the variability of the non-hydrolyzed, class-intact lipid species in milk (6, 16) and their determining factors. To date, the lipid composition among the three major Asian ethnicities living in Singapore has been examined in only one previous study (17), which focused exclusively on fatty acid composition. To our knowledge, no study has systematically assessed how multiple maternal and child factors associate with the HM lipidome at subclass and species levels.

We investigated the associations of a broad range of maternal and infant factors, spanning biological, lifestyle and socioeconomic domains, with the intact HM lipidome at class and species levels in a multi-ethnic Asian cohort. This allowed us to examine whether HM variation would reflect maternal and infant influences not evident from fatty acid composition alone. Identifying such factors may help inform future studies of modifiable influences and their relevance to offspring health.

## Materials and methods

This was an observational GUSTO (Growing Up in Singapore Towards healthy outcomes) cohort study with cross-sectional analyses at 3-weeks and 3-months postpartum HM samples.

### Participants and recruitment

All study procedures were approved by the National University of Singapore Institutional Review Board (NUS-IRB: 2021-810). Participants provided written informed consent. GUSTO recruitment occurred before 14 weeks’ gestation between 2009 and 2010 at the KK Women’s and Children’s Hospital and National University Hospital in Singapore; full cohort (*n* = 1450 participants) details are published (18). Eligibility included pregnant women aged 18 and above, of Chinese, Malay or Indian heritage, with parents of homogenous ethnic background, carrying singleton pregnancies; mixed marriages were excluded. Women diagnosed with Type 1 diabetes, undergoing chemotherapy, or taking psychotropic medication were excluded.

Within the GUSTO cohort, only a subset of participants (*n* = 355) provided HM at 3 weeks and/or 3 months postpartum for research. Of all milk samples collected (*n* = 461), only full-expression HM samples (*n* = 303) were initially considered. For the current study, 8 participants who delivered preterm (<37 weeks) were excluded (*n* = 7 at 3 weeks and *n* = 3 samples at 3 months) as milk composition is known to differ substantially from term samples (19), and the numbers were too small for meaningful statistical analysis. The final sample size was determined by the availability of fully expressed HM samples and study eligibility criteria, as described. After exclusion, there were 293 samples from 228 participants (*n* = 189 at 3 weeks, *n* = 104 at 3 months), with 65 participants providing samples at both timepoints. The flowchart can be found in Supplementary Figure 1.

### Data collection and derivation of variables

Maternal sociodemographic and lifestyle information including age, pre-pregnancy BMI (ppBMI) derived from self-report pre-pregnancy weight and measured height, ethnicity, education, parity, and physical activity before and during pregnancy were collected prospectively at 26–28 weeks’ gestation through interviewer-administered questionnaires. Asian cutoffs were used to categorize ppBMI: underweight (<18.5 kg/m^2^), normal (18.5–22.9 kg/m^2^), overweight (23.0–27.4 kg/m^2^), and obese (≥27.5 kg/m^2^) (20). Physical activity was assessed using metabolic equivalent task (MET) values based on WHO recommendations (21). Tobacco smoke exposure was categorized based on a combination of self-reported smoking, environmental tobacco exposure and plasma cotinine measurements at 26–28 weeks’ gestation (22). Validated questionnaires of State-Trait Anxiety Inventory (State and Trait) were self-administered at 26–28 weeks’ gestation. The STAI-State subscale reflects anxiety at the time of assessment, whereas the STAI-Trait subscale reflects a more stable predisposition toward anxiety. Values ≥ 41 on each subscale were used as cut-offs to determine antenatal maternal anxiety (23, 24). Gestational weight gain (GWG) z-scores were calculated using maternal weights measured in early (4–13 weeks’ gestation) and late pregnancy (30–40 weeks’ gestation), and categorized based on recommendations from the Institute of Medicine (25).

Dietary intake was assessed at 26–28 weeks’ gestation and again during the confinement period (covering the first postpartum month) using a 24-h recall and 3-days food diaries. Women provided detailed descriptions of their food/beverages across two weekdays and one weekend day, along with portion images. Derivation of the postpartum confinement dietary patterns (26) have been published. The confinement diets for 490 women (part of the larger GUSTO cohort) were categorized into 4 patterns – Traditional Chinese confinement diet (TCC), Traditional Indian confinement diet (TIC), Eat-out diet, and Soup-Vegetables-Fruits (SVF) diet (26). Every participant received an adherence score per dietary pattern. Fish oil supplement intake was ascertained at 3 weeks postpartum.

Infant sex and delivery details were extracted from medical records. Birthweight percentiles were derived using the method described by Mikolajczyk et al. (27). Interviewer-administered questionnaires on breastfeeding practices were conducted at 3-weeks and 3-months after delivery. Exclusively and predominantly breastfed infants were combined into one category. This group comprised infants who received no non-HM or solids. In addition to HM, infants in this category may have received certain liquids (water or water-based drinks and fruit juices), drops and syrups (of vitamins, minerals and medicines) (28).

Detailed categorizations for all variables are listed in Table 1.

**TABLE 1:** Participant characteristics by timepoint.

	Summary by timepoint	
Variable	3 weeks *N* = 189^1^	3 months *N* = 104^1^
Maternal age		
18–26	44 (23%)	19 (18%)
27–34	112 (59%)	61 (59%)
≥35	33 (17%)	24 (23%)
Ethnicity		
Chinese	137 (72%)	84 (81%)
Malay	29 (15%)	10 (9.6%)
Indian	23 (12%)	10 (9.6%)
Maternal education (highest)		
University education	95 (50%)	63 (61%)
Non-university education	94 (50%)	40 (39%)
Pre-pregnancy BMI (Asian, kg/m2)		
Normal (18.5–22.9)	100 (55%)	60 (63%)
Underweight (<18.5)	20 (11%)	11 (12%)
Overweight/obese (≥23)	61 (34%)	24 (25%)
Parity		
Nulliparous	91 (48%)	52 (50%)
Parous	98 (52%)	52 (50%)
Tobacco smoke exposure		
No exposure	115 (64%)	71 (74%)
Self-reported exposure (no detectable plasma cotinine)	46 (26%)	20 (21%)
Biomarker plasma cotinine-confirmed exposure	18 (10%)	5 (5.2%)
Physical activity before pregnancy (MET/week)a		
<600	38 (20%)	23 (23%)
600–3000	105 (56%)	57 (56%)
3000+	45 (24%)	21 (21%)
Physical activity during pregnancy (MET/week)		
<600	65 (35%)	46 (45%)
600–3000	93 (49%)	38 (37%)
3000+	30 (16%)	18 (18%)
STAI-S at 26 weeks		
STAI-S < 41	146 (81%)	81 (83%)
STAI-S ≥ 41	35 (19%)	17 (17%)
STAI-T at 26 weeks		
STAI-T < 41	139 (77%)	72 (73%)
STAI-T ≥ 41	42 (23%)	26 (27%)
Gestational weight gain (IOM)b		
Excessive	67 (38%)	30 (32%)
Normal	68 (38%)	43 (45%)
Inadequate	43 (24%)	22 (23%)
Mode of delivery		
Non-labor C-section	22 (12%)	12 (12%)
Intrapartum C-section	24 (13%)	12 (12%)
Vaginal	143 (76%)	80 (77%)
Gestational age at birth (weeks)	39.09 (0.98)	39.16 (0.96)
Infant sex		
Female	94 (50%)	48 (46%)
Male	95 (50%)	56 (54%)
Birth size/weightc		
Appropriate for gestational age (AGA)	138 (73%)	79 (76%)
Small for gestational age (SGA)	17 (9.0%)	4 (3.8%)
Large for gestational age (LGA)	34 (18%)	21 (20%)
Exclusive/predominantd BF (3 weeks)		
Not	124 (66%)	–
Yes	63 (34%)	–
Exclusive/predominant BF (3 months)		
Not	–	57 (55%)
Yes	–	47 (45%)

^1^Mean (SD); *n* (%).

^a^Physical activity measured in metabolic equivalent task (MET) per week as defined by WHO (21).

^b^Gestational weight gain defined as recommended by the Institute of Medicine (25).

^c^Birth size defined according to Mikolajczyk et al. (27).

^d^Exclusive or predominant breastfeeding defined as no non-human milk or solids; may have consumed water, fruit juices, drops, and syrups (vitamins and minerals) as published in Pang (28). BMI, body mass index; BF, breastfeeding; C-section, Cesarean section; STAI-S/T, State–Trait Anxiety Inventory (State/Trait). Full table with NA counts are provided in the Supplementary Table 2.

### Human milk collection and processing

At both timepoints, mothers were requested to fully express milk from one breast between 6.00 and 10.00 a.m., to control for circadian differences, and instructed to wait at least 2 h before emptying that breast, either by manual expression or use of their own breast pump. Meal intake and timings were not systematically recorded. Samples were stored in the fridge until a home visit (within 24 h) then warmed up to 38 °C, gently mixed to ensure homogenization, and divided into 2 mL aliquots. Samples were then transported on ice back to the laboratory where they were stored at −80 °C. Subsequently, samples underwent one freeze-thaw cycle in order to generate 10 μL aliquots.

### Lipidomic analysis

180 μL of chilled ammonium bicarbonate and 810 μL of methyl-tert-butyl-ether/methanol (MTBE/MeOH 7:2 v/v) containing an internal standard (IS) mix (See Supplementary Table 1 for IS concentrations; 10 internal standards were used) was added to 10 μL of HM. Samples were vortexed for 30 s, sonicated for 30 min and centrifuged. The top organic layer (550 μL) was removed to a fresh 96-well plate via an automated liquid handling system (BRAVO, Agilent), dried and reconstituted with 200 μL chloroform/methanol (1:1 v/v) (Portion A). The 2-phase lipid extraction protocol and the instrument workflow have previously been validated and published **(6)**. Neutral lipids – triacylglycerols (TGs) and diacylglycerols (DGs) - were measured via direct-infusion mass spectrometry (DI-MS); polar lipids – phospholipids (PLs) and sphingolipids (SPs) - were measured via reversed-phase liquid chromatography-tandem mass spectrometry (RPLC-MS/MS).

DI-MS: 50 μL of Portion A was diluted ten-fold with a mixture of isopropanol/methanol/chloroform (4:2:1 v/v) containing 7.5 mM ammonium formate. TGs and DGs were measured in positive mode using direct-infusion via the TriVersa NanoMate (Advion, Biosciences, Ithaca, NY, USA) coupled to a Thermo Q Exactive Plus quadrupole-Orbitrap mass spectrometer. Each sample on the plate was sprayed through nano-electrospray nozzles present on an electrospray ionization chip (ESI) into the MS. Each individual tip and nozzle were used only once eliminating any concern of crossover or contamination. Spray initiated from the NanoMate was allowed to stabilize for 30 s before data acquisition commenced. Samples were kept in a 96-well plate at 22 °C for the entire run. The mass range was set between 400 and 1350 m/z. The precursor scans were acquired for 1 min at a resolution setting of 140,000 (profile; AGC 1E6) and the subsequent product ion scans were acquired for 1.5 min at a resolution setting of 17,500 (centroid; AGC 1E5, normalized collision energy 20; fixed first mass m/z 80) with an inclusion list containing all the masses from 400 to 1350 with 1 Da intervals. NanoMate backpressure was set at 1.25 psi with ionization voltage at 1.25 kV. Lock masses (m/z 536.16536 and 680.4022) were used in full scan mode. Results were processed using LipidXplorer 1.2.7 (29). Lipids were identified by their accurate mass (tolerance of up to 5 ppm for MS1) and tandem MS spectra (specifically, neutral losses corresponding to individual fatty acyls). Isotopic correction was performed within LipidXplorer. The neutral lipids were annotated as sum composition.

RPLC-MS/MS: 0.5 μL of Portion A was injected into an Agilent Zorbax RRHD Eclipse C18 column (2.1 mm × 50 mm), and the polar lipids - phospholipids (PLs) and sphingolipids (SP) - were measured on a 1290 Infinity II LC coupled to an Agilent 6495C QqQ system. A dynamic multiple reaction monitoring (dMRM) method was used to monitor both the precursor and product ion transition for each lipid. Identities of each lipid were determined by analyzing retention time (RT) and specific MRM transitions. Total runtime for LC method was 15.8 min with a column temperature of 40 °C. Mobile phase A comprised 40% acetonitrile and 60% water (10 mM ammonium formate) while mobile phase B comprised 90% isopropanol and 10% water (10 mM ammonium formate). A gradient elution with a flow of 0.4 mL/min was employed. Chromatographic conditions were: 40% A, 60% B for 2 min, increased to 100% B (over 10 min), held at 100% B for 2 min, reduced to 20% B and held for another 1.8 min (6). The source parameters are as follows: gas temperature 250 °C; gas flow 14 L/min; nebulizer 35 psi; sheath gas temperature 250 °C; sheath gas flow 11 L/min; capillary voltage 3500 V.

Raw peak areas for PLs and SPs were generated using MRMkit, an open-source software package designed for large cohort studies (30). Isotopic correction was performed to account for M+3 isotopic effect of SM X:Y on PC X−4:Y−1. The full list of MRM transitions that was used (before QC) can be found in the Supplementary Table 8. SM, PC, PE, PI and PS were annotated at the sum composition level; Cer, Hex1Cer, Hex2Cer, LPC and LPE at the molecular-species level.

Quality control and data processing: For both DI-MS and RPLC-MS/MS workflows, rigorous quality control measures included batch quality controls (BQCs), technical quality controls (TQCs) and extraction blanks. Raw peak areas and intensities of lipids were normalized to internal standards added to samples prior to lipid extraction (Supplementary Table 1). Isotopic correction, batch correction and missing-value imputation were performed, where required. An additional probabilistic quotient normalization was performed (31). Further details on lipid quantification can be found in the Supplementary materials.

Batch quality controls used for GUSTO were not derived from the same cohort. Instead, they were pooled milk samples collected in 2015 from de-identified donors. BQC were extracted together with the cohort and analyzed every 10 study samples.

Technical quality controls were derived by pooling an aliquot of all the extracts within the GUSTO cohort. TQCs for the RPLC-MS/MS analysis were run every 20 samples whereas for direct-infusion, TQCs were analyzed in sequence before and after every batch.

A total of 9 extraction blanks were added to the worklist. Finally, only the lipids adhering to the following QC criteria were retained for quantification: (1) coefficient of variation for both TQCs and BQCs < 20% (LC-MS); <25% (Direct-infusion); (2) Signal-to-noise ratio (S/N) > 10; (3) calculated R^2^ for linearity > 0.85 and (4) ≤20% missingness across samples.

### Maternal genotype [fatty acid desaturase (FADS) variants]

Maternal DNA extracted from blood samples collected at 26–28 weeks’ gestation was genotyped using Illumina Omniexpress plus exome arrays (Illumina, Inc.). Full details of the genotyping analysis and quality control measures have previously been published (32).

For this study, 38 single nucleotide polymorphisms (SNPs) in the FADS gene cluster (FADS1, FADS2 and FADS3) on chromosome 11, located between positions 61569830 and 61656117 were initially assessed. The SNPs with call rates less than 95% or minor allele frequency less than 5% or Hardy-Weinberg equilibrium *P*-value less than 1.00E-06 were excluded from analysis. Only those that passed QC in all three ethnic groups were retained. After QC, we evaluated ethnic differences in allele frequencies using global tests (chi-squared or Fisher’s exact test, as appropriate; Benjamini–Hochberg (BH) adjusted *p* < 0.05). Variants showing significant ethnic differences were carried forward, using pairwise comparisons to characterize allele frequencies between ethnic groups (using chi-squared or Fisher’s exact test). Linkage disequilibrium (LD) between SNPs was calculated using the squared Pearson correlation coefficient (r^2^).

### Statistical analyses

All statistical analyses were performed using R version 4.4.1, using FactoMiner (v2.12) for PCA and base R for linear regression with BH correction. Prior to analysis, normalized lipid data were log2-transformed and z-scaled.

A combined principal component analysis for dimensionality reduction was performed on the 3-weeks and 3-months datasets to capture major axes of variation across all samples. A combined PCA approach provided a common lipid coordinate framework (33, 34) and enabled the direct comparison of how maternal and infant factors associated with the HM lipidome at the two separate timepoints on a unified scale. Concordance between combined PCA and PCAs from individual timepoints was assessed by Pearson correlations of loading vectors, and global similarity was assessed by the Escoufier RV coefficient (35). Detailed analyses are available in the Supplementary materials.

Principal component scores were analyzed on their original PCA score scale; β coefficients represent mean differences (unadjusted for univariate models and adjusted for multivariable models) relative to the reference category. While the study focused only on reporting the first three principal components (PCs), top six identified PCs were examined for significant associations and full analyses can be found in the Supplementary materials. Principal component loadings for individual lipid species were examined to identify the strongest contributors to each PC. The lipids were further categorized and visualized using lipid sub-classes, degree of saturation and chain lengths to aid interpretation of underlying lipid patterns driving the variance of lipids and the associations with predictors.

Univariate regression models were fitted separately at 3 weeks and 3 months to assess associations between maternal/infant predictors and PCs (outcomes). Nominally significant variables (*p* < 0.05) were then jointly included in a multivariable regression model where *p*-values were adjusted for multiple comparisons within each principal component using BH correction. Adjusted R^2^ quantified model variance; unique contributions of individual predictors were also ascertained by iteratively refitting the model after dropping each predictor. For the multivariable models at 3 weeks and 3 months, collinearity between factors was assessed using variance inflation factors (VIF); AdjVIF > 5 was used to indicate collinearity (36), and over-adjustment was avoided by ensuring two collinear factors were not used together. To further investigate the association between antenatal anxiety and HM lipids, we conducted multivariable linear regression for all 237 lipid species. The β coefficients are reported in standardized units; BH correction was applied across the 237 tests and p adj values < 0.1 were considered for this follow-up analysis.

Status of maternal fish oil supplementation at 3 weeks was compared across ethnic groups via Fisher’s exact test. Confinement dietary scores for each confinement pattern were analyzed by ethnicity (Wilcoxon rank-sum and *t*-tests); descriptive statistics are presented as median and interquartile ranges (IQR).

To test associations between FADS variants and the HM lipidome, univariate linear regressions were conducted for each SNP using individual lipids (log2-transformed and z-scaled) as outcomes, with BH correction applied per SNP across lipid tests. Each SNP was coded additively as 0, 1 or 2 copies of the alternate allele. Beta coefficients are reported as the SD change in log2 [lipid] per dosage change of the alternate allele. A BH adjusted *p* < 0.05 was used as the threshold for significance.

## Results

Among the 228 participants (Table 1), mean gestational age was similar at both timepoints (39.1 and 39.2 weeks). Women of Chinese ethnicity comprised 72% at 3 weeks and 81% at 3 months; Malay and Indian were 15% and 12% at 3 weeks and 9.6% each at 3 months. Antenatal maternal anxiety was 19% and 17% across the timepoints; tobacco exposure (confirmed by plasma cotinine) 10% and 5%; and exclusive/predominant breastfeeding rates were 34% and 45%. Compared to the broader GUSTO cohort, participants who provided analyzable milk samples were more likely to be Chinese and university educated while being less likely to have tobacco smoke exposure antenatal anxiety. Full participant characteristics of the analytic subset, including NA counts, are in Supplementary Table 2.

After rigorous QC filtering (See Supplementary materials), 237 lipid species from 12 sub-classes were quantified, including triacylglycerols (TGs), diacylglycerols (DGs), phosphatidylcholine (PC), phosphatidylethanolamine (PE), lysophosphatidylcholine (LPC), lysophosphatidylethanolamine (LPE), phosphatidylserine (PS), phosphatidylinositol (PI), sphingomyelin (SM), ceramide (Cer), monohexosylceramide (Hex1Cer), and dihexosylceramide (Hex2Cer). 101 of the lipids quantified were TGs, there were 10 DGs and the remaining 126 were PLs and SPs. In at least 96% of the samples, TG 52:2 and TG 52:3 were the most abundant species.

### Data-driven principal component analysis to group co-varying lipids

Six PCs cumulatively explained 66.7% of the lipid variation (Supplementary Figure 2); PCs 1-3 collectively explained 46.8% of total variance, and individually accounted for 18.6%, 16.0% and 12.2% of lipid variability, respectively.

PC1 comprised strong positive loadings for various polyunsaturated fatty acid-containing triacylglycerols (PUFA-TGs; Chain lengths: C51-C55 and C52-C56) comprising several degrees of unsaturation (double bond, DB 3-7); and negative loadings for several sphingomyelins (SMs) with ≥40 total carbons (e.g., SM 42:3, SM 40:2, SM 40:3, SM 44:3), 3 DB-containing phospholipids [PLs, e.g., Phosphatidylcholine (PC) 38:3, Lysophosphatidylcholine (LPC) 20:3, Phosphatidylinositol (PI) 38:3], and saturated and monounsaturated P1Cs (e.g., PC 30:0, PC 32:0, PC 32:1; Figure 1A).

**FIGURE 1:**
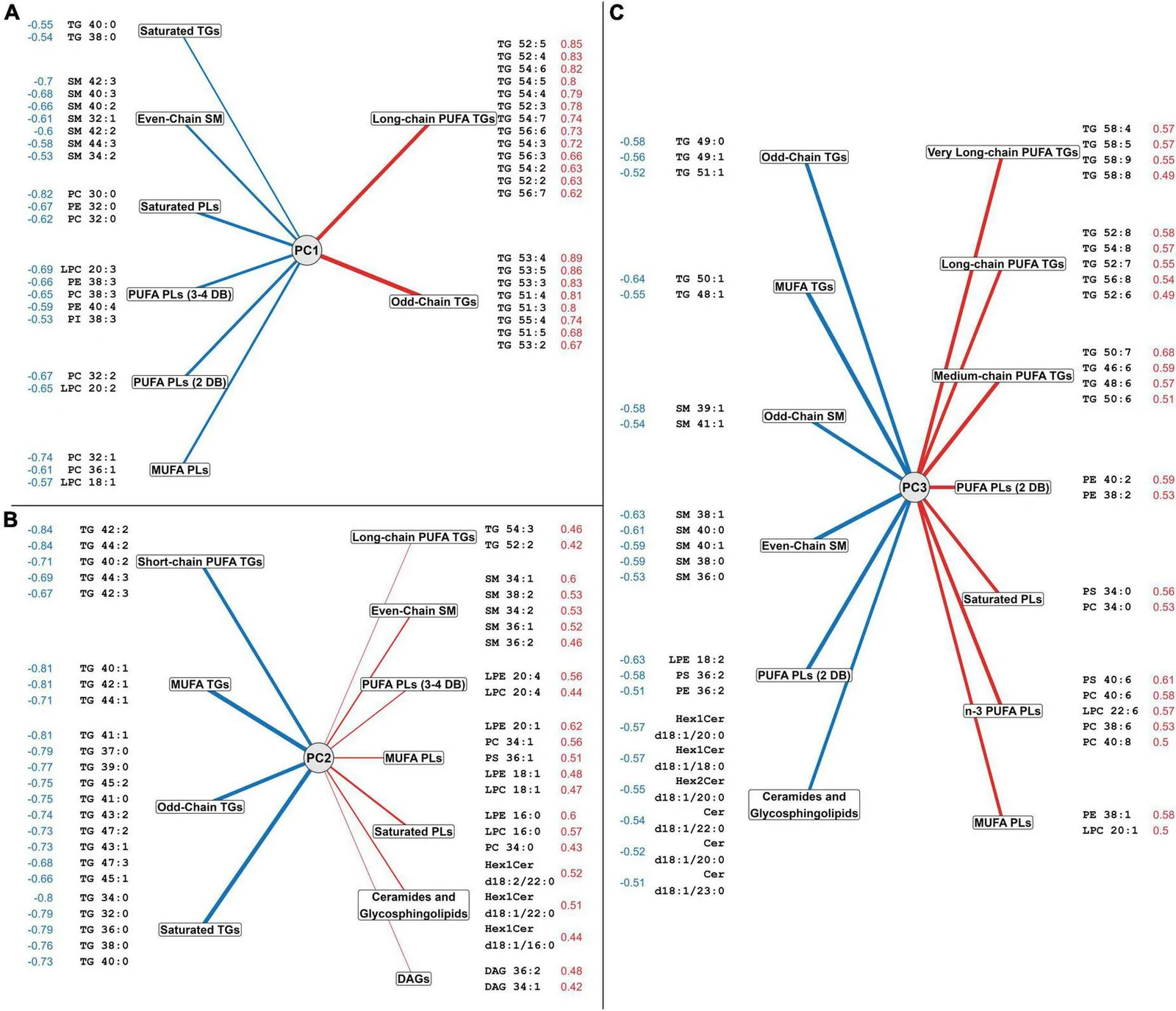
Simplified loading schematics show the top positive and negative loadings of lipid species driving variation in the first three principal components (PCs): **(A)** PC1 (18.6% of variation), **(B)** PC2 (16.0%), and **(C)** PC3 (12.2%). Lipids are classified into specific categories with exact loading values displayed beside each species. Red and blue indicate positive and negative associations, respectively; the width of each line represents median absolute loading of the most strongly associated lipids within that classification. PL, phospholipids; PC, phosphatidylcholine; PE, phosphatidylethanolamine; LPC, lysophosphatidylcholine; LPE, lysophosphatidylethanolamine; PS, phosphatidylserine; PI, phosphatidylinositol; SM, sphingomyelin; DAG, diacylglycerol; TG, triacylglycerol; Cer, ceramide; Hex1Cer, monohexosylceramide; Hex2Cer, dihexosylceramide. MUFA, monounsaturated fatty acids; PUFA, polyunsaturated fatty acids; n-3, omega-3 fatty acids; DB, double bonds.

PC2 was characterized by positive loadings for Lysophosphatidylethanolamine (LPE) 20:4 and LPC 20:4, several monounsaturated PLs [e.g., PC 34:1, Phosphatidylserine (PS) 36:1, LPE 20:1, LPC 18:1] and SMs with C34-C38 carbons (e.g., SM 34:1, SM 34:2, SM 36:1, SM 38:2). The substantial contribution to this component came from the very strong negative loadings, which consisted exclusively of TGs, including odd-chain species (C37-C47, mostly saturated and monounsaturated, e.g., TG 37:0, TG 45:1, TG 43:1) and several saturated and monounsaturated even-chain species (C32-C44, e.g., TG 32:0, TG 38:0, TG 40:1), with a few species containing up to 3 DBs (Figure 1B).

PC3 was dominated by positive loadings of TGs and PLs that comprised ≥6 DBs (e.g., TG 52:8, TG 50:6, PS 40:6, PC 40:6, LPC 22:6, PC 38:6) and negative loadings of several sphingolipids [SPs; e.g., SM 38:1, SM 40:0, monohexosylceramide (Hex1Cer) d18:1/20:0, ceramide (Cer) d18:1/20:0; Figure 1C]. Full loadings are shown in Supplementary Figure 3; concordance of combined PCA with time-specific PCAs is presented in Supplementary Figure 4.

### Maternal factors associated with HM lipid profiles at 3 weeks and 3 months

Univariate associations between 15 maternal and infant factors and the top six identified PCs at 3 weeks were assessed using linear regression models (Supplementary Figure 5). Focusing on the first three PCs, ethnicity was observed to have the strongest associations – Indian vs. Chinese comparison revealed associations across all three PCs, (PC1: β = −5.5, *p* < 0.001; PC2: β = −4.2, *p* < 0.01; PC3: β = −4.0, *p* < 0.001), while the Malay vs. Chinese comparison revealed associations for PC1 and PC3 (PC1: β = −6.8, *p* < 0.001; PC3: β = −5.1, *p* < 0.001). Other associated factors included maternal age (PC2: β = −2.29 for 27–34 vs. 18–26, *p* < 0.05; β = −3.31 for ≥35 vs. 18–26, *p* < 0.05), maternal education (PC1: β = −4.0, *p* < 0.0001), antenatal state-anxiety (STAI-S, PC3: β = −2.2, *p* < 0.01), small-for-gestational-age (SGA, PC3: β = 3.0, *p* < 0.01), and exclusive/predominant breastfeeding (PC1: β = 2.7, *p* < 0.01). Several variables, such as large-for-gestational-age, intrapartum cesarean section, gestational weight gain and infant sex did not associate with any of the PCs (Supplementary Figure 5).

**FIGURE 2:**
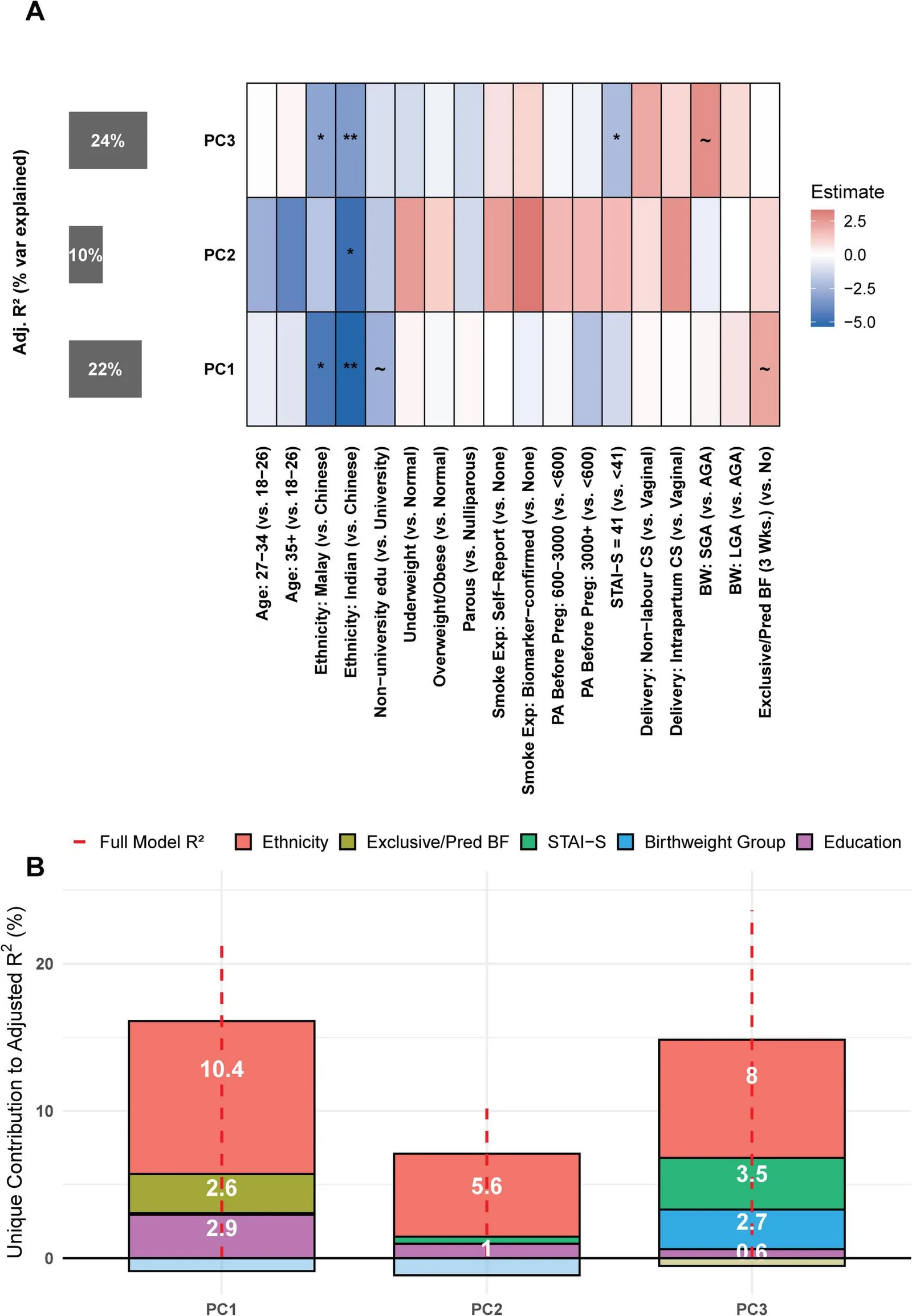
**(A)** The heatmap displays multivariable linear regression associations between selected factors (identified in univariate analyses) and PC1–PC3 at 3 weeks postpartum. The beta coefficients are on the original PCA score scale, representing adjusted mean differences relative to the respective reference groups. Red and blue demonstrate positive and negative associations, with color intensities demonstrating strength of associations. P-adjusted values are represented by asterisks denoting statistical significance (*p adj < 0.05, **p adj < 0.01, ∼p adj < 0.1). Together, the shown predictors explain 22%, 10% and 24% (total adjusted R^2^) of the variance in PC1–PC3 respectively. **(B)** Stacked bar-chart illustrates the unique contribution (adjusted % R^2^) of individual factors, such as ethnicity, maternal education, antenatal maternal anxiety (STAI-S), birthweight and breastfeeding on PC1–PC3 at 3 weeks. Corresponding plots for PCs 1–6 are provided in Supplementary materials.

Eleven key factors from the univariate analysis were subsequently included in a multivariable linear regression model (Figure 2A). After correction for multiple comparisons, associations with ethnicity and STAI-S remained. The Indian vs. Chinese comparison remained inversely associated with the first three PCs (Figure 2A; PC1: β = −5.3, 95% CI: −8.0, −2.7, p adj < 0.01; PC2: β = −5.0, 95% CI: −7.9, −2.0, p adj < 0.05; PC3: β = −3.4, 95% CI: −5.3, −1.5, p adj < 0.01), while Malay vs. Chinese comparison remained inversely associated with PC1 (β = −4.6, 95% CI: −7.5, −1.7, p adj < 0.05) and PC3 (β = −3.1, 95% CI: −5.1, −1.0, p adj < 0.05). STAI-S remained inversely associated with PC3 (β = −2.2, 95% CI: −3.8, −0.6, p adj < 0.05).

Associations for maternal education, exclusive/predominant breastfeeding and SGA with the PCs were weakened (p adj < 0.1) but directionally consistent with the univariate analyses (Figure 2A). The other predictors no longer showed associations in the multivariable context. Together, the 11 variables included in the multivariable model explained 22%, 10% and 24% (adjusted R^2^) of the variance in PC1-PC3, respectively (Figure 2A). Ethnicity uniquely explained 10.4%, 5.6%, and 8.0% of the total variance in the first three PCs, effectively accounting for 47%, 55% and 34% of the model-explained variance (Figure 2B).

Similar analysis at 3 months (Supplementary Figures 8, 9, respectively), revealed fewer and weaker associations with maternal and infant factors compared to 3 weeks. Nevertheless, (Supplementary Figure 8), ethnicity, again, demonstrated the strongest associations, with patterns parallel to those observed at 3 weeks (Figure 2A, Supplementary Figure 8): in multivariable analyses (Supplementary Figure 9), Indian vs. Chinese retained its association with PC2 (β = −8.4, 95% CI: −12.3, −4.5, p adj < 0.001). Indian ethnicity had a similar effect size on PC1 (β = −3.8, 95% CI: −7.7, −0.04), PC3 (β = −2.5, 95% CI: −5.2, 0.2), and Malay ethnicity on PC1 (β = −4.7, 95% CI: −8.6, −0.8), PC3 (β = −1.7, 95% CI: −4.4, 1.1), although the adjusted *p*-values did not reach statistical significance. Together, all 7 variables in the multivariable model explained 19%, 30% and 20% of variance in the first three PCs, respectively. Ethnicity explained 7.4%, 15.6%, and 2.6% of total variance of PC1, PC2 and PC3 respectively, effectively accounting for 38%, 52% and 13% of model-explained variance (Supplementary Figure 10).

### Ethnic differences in human milk lipid profiles

#### PUFA-rich milk lipid profile in Chinese ethnicity

The strong inverse associations of Malay and Indian compared to Chinese across PC1 and PC3 indicate distinct lipid profiles (Figure 2A, Supplementary Figure 9): both PCs load positively to several PUFA-containing lipids – including TGs [both odd and even-chain, medium-chain, long-chain (C52-57) and very long-chain (≥C58) variants] and phospholipids [phosphatidylethanolamine (PE), PC, LPE, LPC, PS; Figure 1] – consistent with higher levels of PUFA-containing lipids observed in the milk of Chinese mothers at both 3 weeks and 3 months compared to Malay and Indian.

Because ethnicity and PUFA-containing lipids showed strong associations, we undertook additional exploratory analyses to examine whether fish oil supplementation, Asian confinement dietary patterns, and FADS1/FADS2 variants might help contextualize the observed differences.

Given that maternal supplementation with docosahexaenoic acid (DHA) and other n-3 PUFAs increases their levels in HM (37, 38), we first examined fish oil supplementation as a potential explanation for the observed ethnic differences. At 3-weeks postpartum, only a small proportion of mothers reported fish oil supplementation (Chinese: 18.2%, Malay: 15.2%, Indian: 16%), with no significant differences across ethnic groups (global Fisher’s exact test, *p* = 0.73, Supplementary Figure 11). Regression analysis excluding participants on fish oil supplements yielded similar results (data not shown) with Chinese mothers still demonstrating higher levels of PUFA lipids compared to their counterparts. This suggests that other sources of PUFA variation associated with these ethnicities are present within the GUSTO cohort.

**FIGURE 3:**
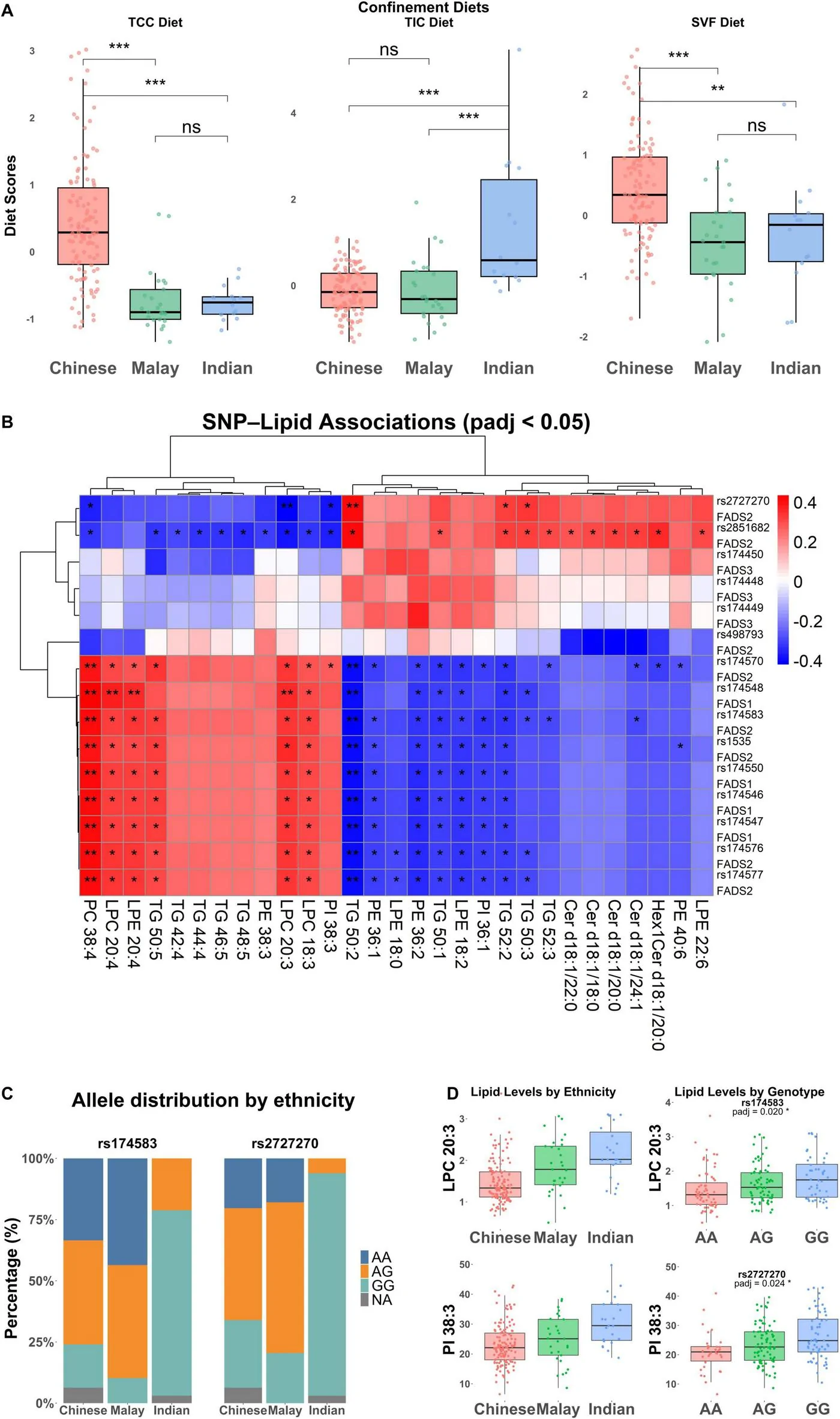
Plots show ethnic differences in terms of confinement dietary patterns, genetic distributions and associated lipid-genotype relationships. **(A)** Varying confinement dietary patterns across ethnicities: Soups-Vegetables-Fruits (SVF) confinement diet differs significantly between Chinese and Malay (*p* < 0.0001), and Chinese and Indian (*p* < 0.01), but not between Malay and Indian (*p* = 0.625); Traditional Chinese confinement (TCC) diet differs significantly between Chinese and Malay (*p* < 0.0001), and Chinese and Indian (*p* < 0.0001), but not Malay and Indian (*p* = 0.671); and the TIC diet shows significant differences between Chinese and Indian (*p* < 0.0001), and Malay and Indian (*p* < 0.001), but not Chinese and Malay (*p* = 0.589) **(B)** Heatmap showing univariate SNP-Lipid associations after BH correction, specifically displaying only those lipids that have at least one significant association (p adj < 0.05). Red indicates positive associations, and blue, negative. Beta estimates are per-allele additive and reported in SD change in log2 lipid concentrations per dosage change of alternate allele. **(C)** Stacked bar chart shows the rs174583 and rs2727270 allele distribution by ethnicity. For rs174583 (Ref/Alt: A/G), Chinese and Malay have alternate allele frequencies of 41.5% and 25.9%, respectively while Indian showed a markedly higher alternate allele frequency of 88.6%. For rs2727270 (Ref/Alt: G/A), Chinese and Malay have comparable alternate allele frequencies (46.5% and 51.7%, respectively), while Indian had an alternate allele frequency of 2.2%. Pairwise ethnicity comparisons are provided in the Supplementary materials. **(D)** Box plots show normalized LPC 20:3 and PI 38:3 concentrations across ethnic groups and rs174583 and rs2727270 genotypes. Higher levels of LPC 20:3 were observed in individuals with increased dosage of the G allele at rs174583, whilst lower levels of PI 38:3 were observed in those with increased A-allele dosage at rs2727270. At both loci, Indians were more commonly observed to have the GG genotype. *p < 0.05, **p < 0.01, ***p < 0.001

We then examined whether the ethnic differences could alternatively be attributed to traditional confinement diets (followed in the first postpartum month). Four distinct dietary patterns were identified within the GUSTO cohort (26) and these were the Traditional Chinese Confinement (TCC) diet, the Traditional Indian Confinement (TIC) diet, the Soups-Vegetables-Fruits (SVF) diet, and the Eat-Out diet. The TCC diet was generally characterized by traditional dried fruits, herbal tea, Chinese herbs, sesame oil, and food cooked with wine or alcohol, while the TIC was characterized by Indian herbs, ethnic breads, whole milk and dairy derivatives, such as butter and ghee. The SVF diet comprised of vegetables, meat, seafood, fish, fruits and was inversely associated with milk-based beverages. The Eat-Out diet was typical of fast-food items, such as pizzas, burgers, carbonated beverages and fried potatoes (26). Chinese mothers scored higher than their ethnic counterparts on two diets (Figure 3A): the SVF diet (median = 0.34; IQR: −0.12 to 0.96) compared to Malay (median = −0.44; IQR: −0.97 to 0.05; *p* < 0.0001) and Indian (median = −0.15; IQR: −0.76 to 0.03; *p* < 0.01), and the TCC diet (median = 0.29; IQR: −0.19 to 0.96) compared to Malay (median = −0.90; IQR: −1.00 to −0.56; *p* < 0.0001) and Indian (median = −0.75; IQR: −0.93 to −0.67; *p* < 0.0001). Notably, the SVF diet, rich in fish and seafood, while the TCC diet, rich in ingredients such as sesame oil, both potentially provide sources of PUFAs (26). There was no significant difference in either of these diets between Malay and Indian (SVF, *p* = 0.6; TCC, *p* = 0.67; Figure 3A).

#### Distinctive lipid signatures in Malay and Indian ethnicities: sphingolipids, low-unsaturation and odd-chain lipids

The inverse associations of Malay and Indian compared to Chinese across PC1 and PC3 (Figure 2A, Supplementary Figure 9) indicated that the milk of Malay and Indian mothers contained more SPs, particularly SM ≥ 40 total carbons, and both PLs and TGs with low degrees of unsaturation (even-chain, 0-3 DB). These lipids corresponded to the negative loadings in PC1 and PC3 (Figure 1).

PC2, a distinct observation associated only with the Indian vs. Chinese comparison (Figure 2A), reflected the higher amounts of several odd and even-chain TGs that were mostly saturated and monounsaturated (with a few 2-3 DB species) in Indian compared to Chinese. The TG total carbon number was <47, which is representative of short to medium chain TGs. In contrast, the 20:4 fatty acid-containing LPE and LPC (LPE 20:4, LPC 20:4) were lower in Indian compared to Chinese (Figure 1). Additionally, other odd-chain lipids, such as SM 39:1, SM 41:1, TG 49:0, and TG 51:1, were observed at higher levels in Indian compared to Chinese.

Indian mothers scored significantly higher on the TIC diet (median = 0.59; IQR: 0.21–2.46) compared to Malay (median = −0.31; IQR: −0.64 to 0.34; *p* < 0.001) and Chinese (median = −0.15; IQR: −0.51 to 0.29; *p* < 0.001) mothers, which featured dairy-rich components, like whole-milk, and derived items like butter and ghee (26), and are known to elevate the presence of odd-chain lipids in blood (39, 40). There were no significant differences in TIC scores between Malay and Chinese (*p* = 0.58; Figure 3A). Similarly, the Eat-Out diet was not distinguishing for any ethnicity (data not shown).

#### HM lipids and FADS genotypic variants

Fatty acid desaturases (FADS) are enzymes that introduce double bonds into fatty acid chains; their polymorphisms have been reported to modulate PUFA levels in human blood and tissues (41). We examined whether FADS variation might contribute to observed ethnicity-related differences. After QC and linkage disequilibrium (LD) grouping, we prioritized 2 representative SNPs (Figures 3B–D, Supplementary materials): rs174583 and rs2727270.

At rs174583, Indians had a significantly higher alternate allele (G) frequency (88.6%), compared to 41.5% in Chinese and 25.9% in Malays (Figure 3C). In contrast, at rs2727270, Indians had a much lower alternate allele (A) frequency (2.2%) compared to 46.5% in Chinese and 51.7% in Malays (Figure 3C). Pairwise genotype comparisons by ethnicity are shown in Supplementary Table 5. These frequency distributions may underlie the observed ethnic differences in n-3 and n-6 lipid profiles.

SNP-lipid analysis at rs174583 showed that with higher G-allele dosage, individuals had higher levels of LPC 20:3 (per dosage unit β = 0.35, CI: 0.16–0.53, p adj = 0.024) compared to those with the reference homozygote, AA (observed more in Chinese and Malays; Figure 3D). For rs2727270, those with higher A-allele dosage had lower levels of PI 38:3 (per dosage unit β = −0.37, CI: −0.57 to −0.17, p adj = 0.020) compared to those with reference GG genotype (predominant among Indians; Figure 3D).

At rs174583, the G allele was also associated with higher levels of LPC 20:4 and LPE 20:4, and lower levels of 18:2-containing lipids, such as LPE 18:2 and PE 36:2 (Figure 3B). Despite this genetic association, in the multivariable analyses (Figure 2), Indians had lower levels of LPC 20:4 and LPE 20:4, and higher levels of LPE 18:2 and PE 36:2, compared to Chinese.

### Antenatal maternal anxiety and HM lipid profiles

**FIGURE 4:**
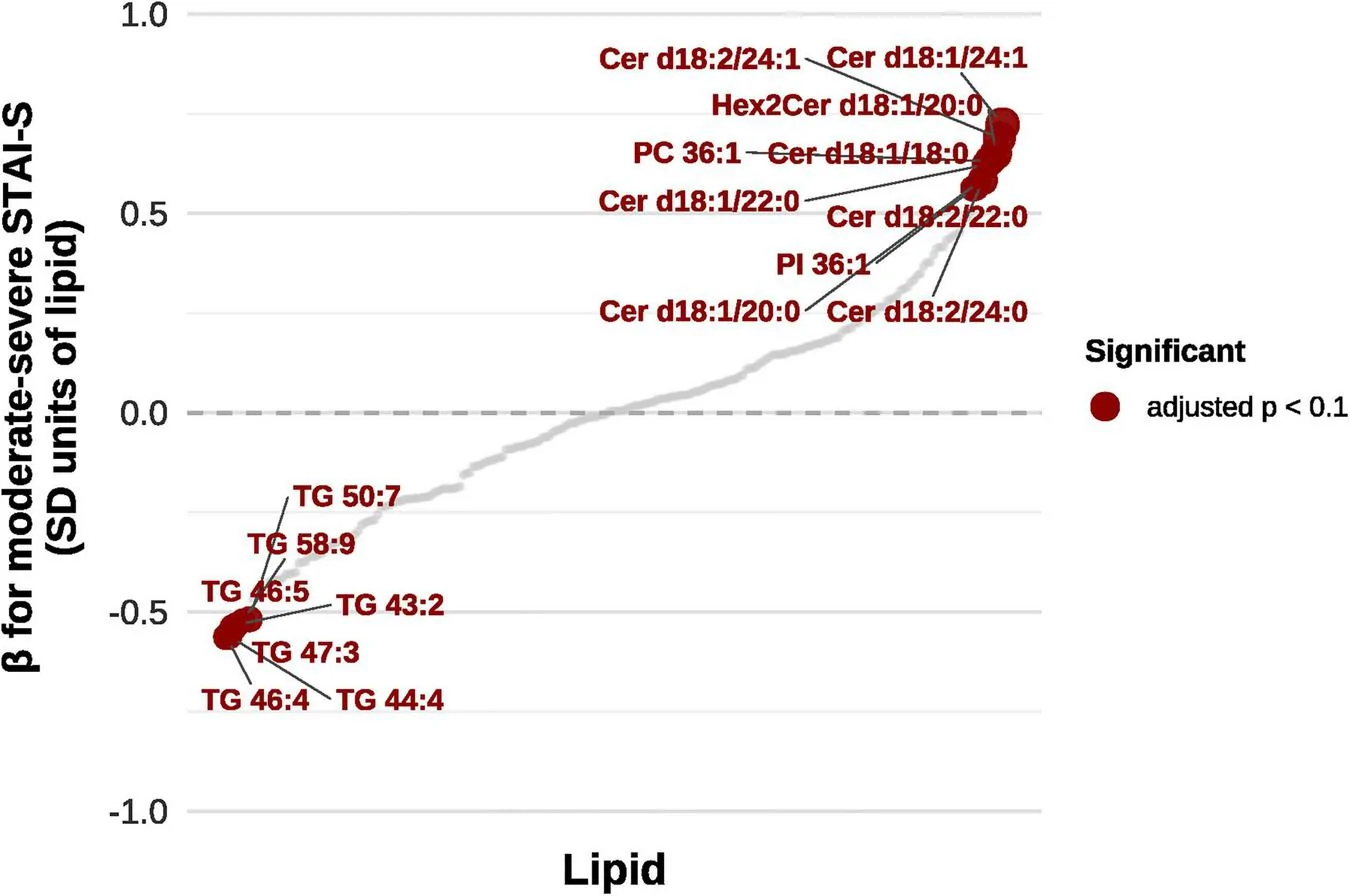
Effect-Size dot plot displays beta-estimates from the multivariable regression analysis comparing moderate-severe antenatal anxiety (STAI-S ≥ 41) with lower antenatal anxiety (Reference group: STAI-S < 41) across all 237 lipids. Higher beta-estimates indicate lipid species that are elevated in participants experiencing moderate-severe anxiety at 26 weeks of pregnancy compared to those with lower anxiety. Beta-estimates are in standardized units. Red dots indicate lipids with BH-adjusted *p*-values < 0.1; dot size corresponds to statistical significance, with larger dots representing smaller *p*-values.

Antenatal maternal state-anxiety (STAI-S scores) showed a specific and transient association with PC3 – differences were apparent at 3 weeks, but not 3 months. Since ethnicity explained a larger proportion of the variation of PC3 than STAI-S (8.0% vs. 3.5%; Figure 2B), we conducted an additional multivariable regression. Adjusting for the same 11 predictors as in the 3-weeks multivariable analysis and with FDR correction, we identified 17 lipids that were associated with STAI-S (p adj < 0.1) (Figure 4).

Among these, mothers experiencing moderate-severe anxiety were observed to have higher levels of 7 ceramides (β = 0.56–0.72), including Cer d18:1/24:1 (β = 0.72, 95% CI: 0.32, 1.13, p adj = 0.086) and Cer d18:2/24:1 (β = 0.69, 95% CI: 0.28, 1.09, p adj = 0.086). Hex2Cer d18:1/20:0 (β = 0.65, 95% CI: 0.26, 1.04, p adj = 0.086), and monounsaturated PLs, including PC 36:1 (β = 0.63, 95% CI: 0.23, 1.03, p adj = 0.086) and PI 36:1 (β = 0.56, 95% CI: 0.17, 0.95, p adj = 0.086) were also found to be higher. Several TGs, including species with 6 or more DBs such as TG 50:7 (β = −0.52, 95% CI: −0.87, −0.16, p adj = 0.086) and TG 58:9 (β = −0.52, 95% CI: −0.89, −0.15, p adj = 0.086), were found to be lower in more anxious women, plausibly suggesting a reduction in DHA levels. Notably, SPs made up 80% of the elevated lipids (8 out of 10), suggesting potential alterations in sphingolipid metabolism.

## Discussion

The HM lipidome appeared more variable at 3 weeks than at 3 months postpartum with ethnicity showing consistent associations at both timepoints. Antenatal maternal anxiety, as measured by STAI-S scores, was associated with HM lipid profiles at 3 weeks, but not 3 months. Other variables were also found to have weaker associations. These attenuated associations should be interpreted cautiously due to greater cohort homogeneity and smaller sample sizes at this time point; subgroup numbers for variables, such as ethnicity and STAI-S were also lower at 3 months. Nevertheless, ethnicity-related patterns at 3 months were directionally similar to those observed at 3 weeks, and attenuation in statistical significance may reflect lower precision rather than a complete lack of differences. Overall, the factors analyzed at 3 weeks and 3 months explained only part of the HM lipidome variation, leaving additional determinants to be identified.

### Ethnicity and HM lipid composition

Ethnicity is a complex multifactorial construct; our findings suggest that ethnic-based variations among the three major ethnic groups in Singapore may reflect multiple contributing factors, which may help inform future studies.

Variations in postpartum diets and genetic variants of FADS1 and FADS2 may help contextualize the observed differences in HM lipids. Chinese women had higher concentrations of PUFA-TGs, which may be consistent with greater consumption of PUFA-rich foods present in TCC/SVF diets, whereas Indian women exhibited a more distinctive odd-chain profile (particularly in the saturated and monounsaturated species across TG, PL and SP classes), which may be consistent with their higher intake of dairy and derived items. GUSTO researchers examining plasma fatty acids in a larger cohort at 26–28 weeks’ gestation found similarly higher levels of n-3 PUFA - DHA, DPA (22:5), and EPA (20:5)- in Chinese participants compared to Malay and Indian (in phosphatidylcholine only), suggesting these differences may reflect longer-term dietary patterns (32). Differences in sphingolipid levels remained unexplained and could not be attributed to the factors studied. Researchers previously observed no difference in SM/Cer levels in milk of women with varying levels of sphingolipid intake, hypothesizing that sphingolipid metabolism in the mammary gland may be tightly regulated and resistant to dietary influences (42).

To date, only one study has examined ethnic associations in HM fatty acids within a local Singaporean context (17). They observed that Indians had higher levels of n-6 fatty acids compared to Chinese and Malay, and that Chinese had significantly higher DHA compared to their ethnic counterparts, yet no significant differences in the n-3 and n-6 consumption were identified. This suggested that other maternal factors, beyond diet, may contribute. Our findings are consistent with this possibility: that confinement dietary patterns and FADS variation are plausible contributors to observed ethnic differences.

The A allele at rs174583 has previously been associated with lower levels of long-chain n-6 fatty acids (i.e., arachidonic acid, FA 20:4) and higher levels of 18:2 in plasma phospholipids during pregnancy and in milk (41, 43, 44). This aligns with our SNP observations: the homozygous GG genotype (predominant in Indians) at rs174583 was associated with lower levels of 18:2-containing lipids and higher 20:4-containing lipids in the SNP analyses compared to AA and AG genotypes, whereas the direction of some of these associations was reversed in our multivariable comparisons. This may highlight the susceptibility of some of these lipids to modification by dietary trends, environmental exposures and other plausible factors (45). One plausible explanation is that Indian mothers’ adherence to the TIC diet, low in seafood/fish and high in dairy (i.e., higher saturated and monounsaturated fat) may have provided limited dietary sources of the more long-chain 20:4-containing lipids whereas Chinese mothers’ consumption of fish-rich diets (SVF/TCC) may have supplied preformed PUFAs more directly. Specific lipids, such as LPC 20:3, LPC 18:3 and PI 38:3 (most plausibly containing the n-6 18:3 or 20:3 fatty acids) were elevated in Indians in both our SNP-lipid analyses and multivariable comparisons, suggesting that these lipids may be less perturbed by co-exposures.

### Antenatal anxiety and HM lipid composition

To our knowledge, this is the first study to report elevated ceramides in the mature milk of mothers with moderate to severe state anxiety during pregnancy. We also observed lower levels of several PUFA-containing TG species in mothers with anxiety, including TGs with 6 or more DBs. These differences were observed at 3 weeks but not at 3 months, possibly because the STAI-S assessed at 26–28 weeks’ gestation was temporally closer to the 3-weeks samples than to the 3-months samples; smaller sample size and reduced variability of the HM lipidome at 3 months may also have attenuated this link. Literature in pregnant populations suggests that antenatal STAI-S may be more sensitive in capturing pregnancy-related anxieties compared to STAI-T (46, 47) and may therefore explain why the former and not the latter was associated with the early postpartum HM lipidome.

The idea that maternal psychological state may be linked with biologically meaningful signals relevant to the infant is not new. Randomized controlled trials across different lactation cohorts, including Asian cohorts from Malaysia and China and a UK cohort, have shown that relaxation interventions during lactation are associated with lower maternal stress physiology, such as lower salivary cortisol, and improved infant outcomes, including longer sleep duration, less crying, greater weight gain, as well as changes in the HM microbiome (48–51). Together, these findings are consistent with a plausible link between maternal psychology and measurable lactation-related and infant outcomes.

Our findings are consistent with previous research showing elevation of ceramides in various mental health disorders, including depression, schizophrenia, bipolar and anxiety disorders, in plasma and brain white matter, suggesting possible dysregulated sphingolipid metabolism (52–54). Similarly, low plasma PUFA levels have been associated with anxiety and depressive disorders, with n-3 PUFAs inversely associated with mental health symptoms (55–57).

While these relationships are more established in plasma studies, evidence demonstrating altered n-3 and n-6 fatty acid levels in HM among individuals with anxiety/depressive conditions is inconsistent. Increased antenatal depressive symptoms before 20 weeks of gestation was reportedly associated with lower DHA levels in mature milk (58), but not in early postpartum samples (59), suggesting that lactation stage may also play a crucial role.

How maternal anxiety could potentially be associated with HM lipids remains unclear and requires further investigation. In adults, elevated plasma ceramides have been linked to increased cellular stress and dysregulated signaling pathways (52, 60). It is shown that in adults with depression and anxiety, acid sphingomyelinase activity is elevated (61), catalyzing the conversion of SM to Cer; notably, some antidepressant drugs exert their therapeutic effects partly through inhibiting sphingomyelinase activity (62, 63). Whether such increased sphingomyelinase activity occurs in the mammary gland in association with maternal anxiety remains to be investigated.

### Potential impact of HM lipid composition on infant outcomes

Differences in HM lipids may be associated with infant and child outcomes, although reference ranges and the biological significance of variation in specific HM lipids remain incompletely defined (64). Long-chain PUFAs, both n-3 and n-6 fatty acids, comprise essential and conditionally essential fatty acids and have long been known to be indispensable to human health. They are important for neural, retinal, brain, and cognitive development of infants (65, 66); are integral to maintaining cell structure and fluidity, and serve as precursors to bioactive factors, such as eicosanoids that control critical cellular processes (67). Long-chain PUFA supplementation has shown inconsistent effects on language and psychomotor development, but more consistent effects have been observed for infant visual attention and problem solving (66).

As for the odd-chain fatty acids, their biological significance with relation to early-life nutrition remains poorly characterized. In adults, studies show that higher levels of circulating odd-chain fatty acids increase membrane fluidity more than PUFAs and correlate with a lowered disease risk (e.g. chronic inflammation, Type 2 diabetes, cardiovascular disease) (68–70). Less is known about their role in infant health, although recent evidence in animal models points toward anti-inflammatory effects in the gut (71).

It is unclear whether the observed changes in ceramide concentrations have meaningful implications for infant health, thus requiring further investigation. While anxiety and depression during pregnancy have been linked to numerous unfavorable offspring outcomes (72–74), the underlying mechanisms are unclear; our findings suggest that HM lipid alterations may be one possible contributor. Longitudinal studies are required to assess infant outcomes from altered intake of HM lipids and to establish clinical significance.

Observed fold-changes in lipid levels may seem modest, but several considerations add to their biological relevance. HM is the primary source of nutrition for infants, one that is sometimes consumed every few hours for several months through to the first 1–2 years of their lives. Modest amounts from repeated exposures over critical developmental windows may have important implications for offspring health. For example, it is known that LPC-DHA is specifically transported across the blood-brain barrier via the Mfsd2a transporter (75); human infants and children with mutations in Mfsd2a presented with microcephaly and impaired neurodevelopment (76, 77). While such direct evidence is lacking for other HM lipids, certain lipids, such as Cers, have known bioactive roles in adults (52, 60). It is unknown if they have similar bioactivity in infants when consumed in HM, although coordinated changes across multiple lipid classes, such as high Cer and low PUFA lipids, may have a cumulative effect greater than individual changes alone. Longitudinal studies of large cohorts are required to establish clinically relevant levels.

### Strengths and limitations

A key strength of our study is the comprehensive lipidomic profiling, which captured variation in 12 lipid subclasses and 237 lipid species, complementing hydrolyzed fatty acid data, which is prevalent in existing HM literature. The deep phenotyping and data collection in GUSTO enabled us to link HM characterization with genetic data and confinement diets. However, our modest sample size precluded the conduct of a genome-wide association study (GWAS), which might have provided more comprehensive insight into genetic influences on HM lipid composition. Our limited sample size may have also reduced statistical power to detect weaker genetic associations; validation of our FADS finding in larger cohorts is warranted.

Additionally, the confinement data lacked quantitative nutritional assessment of macronutrients, such as total energy and fat intake, limiting our interpretation of associations between diet and HM lipids. Statistical power to test for associations with a number of key factors at 3 months was limited as the sample population was more homogeneous and considerably smaller. Notably, the 3-months cohort had more Chinese and a higher educational profile, with smaller numbers of Malay and Indian participants, limiting the generalizability of findings to more ethnically and educationally diverse populations. Nevertheless, as many ethnic associations, while attenuated, remained significant and directionally consistent with those observed at 3 weeks, this attenuation may be more reflective of reduced power and precision rather than true temporal changes.

## Conclusion

In conclusion, the HM lipidome was most strongly associated with ethnicity, and differences may partly reflect confinement diets and FADS1/FADS2 variants. Ethnic differences persisted across timepoints, with attenuated effect sizes at 3 months. Antenatal maternal anxiety was independently associated with lower levels of PUFA lipids and higher levels of ceramides in HM at 3 weeks postpartum. Further study of culturally relevant nutritional guidance and antenatal mental health in relation to human milk composition is warranted. Future studies should also investigate infant outcomes in relation to HM ceramides, incorporate mechanistic assays and clarify clinically relevant thresholds. This study focused on early postpartum milk samples within a multi-ethnic, urban Singapore context; the extrapolation of these results to other settings or lactational stages may be limited. Replication in independent cohorts with larger sample sizes and balanced ethnic groups is warranted to strengthen the generalizability of the findings.

## Data Availability

The raw data supporting the conclusions of this article will be made available by the corresponding author upon request.
