# Supplementary material for: Ethnicity and antenatal maternal anxiety: associations with the human milk lipidome in a multi-ethnic Asian cohort
Source: Front Nutr. 2026 Aug 20;13:1908654. doi: 10.3389/fnut.2026.1908654 (PMC13538122; doi:10.3389/fnut.2026.1908654)
Supplement: Supplementary file 2 [file Supplementary_file_1.docx]

Supplementary Materials – Ethnicity and antenatal maternal anxiety: influences on the human milk lipidome in a multi-ethnic Asian cohort

Jayashree Selvalatchmanan^1^, Shanshan Ji^1^, Li Chen^2^, Helen Chen^3,7^, Mary F.F. Chong^2,4^, Johan G. Eriksson^2,5,6,9^, Yap-Seng Chong^2,9^, [Mei Chien Chua](https://pubmed-ncbi-nlm-nih-gov.libproxy1.nus.edu.sg/?sort=date&term=Chua+MC&cauthor_id=40938107)^7,8^, Kok Hian Tan^7,8^, David Bradley^10^, Markus Wenk^1,14^, Mary E. Wlodek^2,12,13^, Shiao-Yng Chan^2,9^, Wei Wei Pang^9,11†*^, and Federico Torta^1,15,16†*^

1 Singapore Lipidomics Incubator, Department of Biochemistry, Yong Loo Lin School of Medicine, National University of Singapore, Singapore

2 Institute for Human Development and Potential, Agency for Science, Technology and Research (A*STAR), Singapore

3 Department of Psychological Medicine, KK Women’s and Children’s Hospital, Singapore

4 Saw Swee Hock School of Public Health, National University of Singapore and National University Health System, Singapore

5 Folkhälsan Research Center, Helsinki, Finland

6 Department of General Practice and Primary Health Care, University of Helsinki, Helsinki, Finland

7 Duke-NUS Medical School, Singapore

8 Department of Maternal Fetal Medicine, KK Women’s and Children’s Hospital, Singapore

9 Department of Obstetrics and Gynaecology, Yong Loo Lin School of Medicine, National University of Singapore, Singapore

10 Pacific Technology Marketing, Lucaston, Tasmania, Australia

11 Global Centre for Asian Women’s Health (GloW), Yong Loo Lin School of Medicine, National University of Singapore, Singapore

12 Department of Obstetrics and Gynaecology, The University of Melbourne, Parkville, Australia

13 School of Molecular Sciences, The University of Western Australia, Perth, Australia

14 College of Health and Life Sciences, Hamad Bin Khalifa University, Doha, Qatar

15 Signature Research Program in Cardiovascular and Metabolic Disorders, Duke-NUS Medical School, Singapore

16 Precision Medicine Translational Research Programme and Department of Biochemistry, Yong Loo Lin School of Medicine, National University of Singapore, Singapore

†These authors share last authorship

*Correspondence: [federico.torta@duke-nus.edu.sg](mailto:federico.torta@duke-nus.edu.sg); (F.T.) and [obgpww@nus.edu.sg](mailto:obgpww@nus.edu.sg) (WW.P)

[**Supplementary Figure 1 :** Flowchart detailing the exclusion and inclusion criteria for the selection of human milk samples from the GUSTO (Growing Up in Singapore Towards healthy Outcomes) cohort leading to the final sample numbers used at the 3-week and 3-month postpartum timepoints.	5](#_Toc225866997)

[**Supplementary Figure 2**: Scree plot shows explained variance (%) of each principal component derived from PCA of lipidomic dataset (both three weeks and three months)	14](#_Toc225866998)

[**Supplementary Figure 3**: Simplified path diagrams shows the top positive and negative loadings of lipid species driving variation in PCs 4-6: (A) PC4 (8.8% of variation), (B) PC5 (6.2%), and (C) PC6 (4.4%). Lipids are classified into specific categories with exact loading values displayed beside each species. Red and blue indicate positive and negative associations, respectively; the width of each line represents median absolute loading of the most strongly associated lipids within that classification. Abbreviations: PL, phospholipids; PC, phosphatidylcholine; PE, phosphatidylethanolamine LPC, lysophosphatidylcholine; LPE, lysophosphatidylethanolamine; PS, phosphatidylserine; PI, phosphatidylinositol; SM, sphingomyelin; DAG, diacylglycerol; TG, triacylglycerol; Cer, ceramide; Hex1Cer, monohexosylceramide; Hex2Cer, dihexosylceramide. MUFA, monounsaturated fatty acids; PUFA, polyunsaturated fatty acids; n-3, omega-3 fatty acids; DB, double bonds.	15](#_Toc225866999)

[**Supplementary Figure 4**: Concordance analysis using Pearson’s correlations between vector loadings from combined PCA and three-week (top) and three-month (bottom) datasets. Escoufier’s RV coefficients are displayed to show global subspace similarity between the combined data and the three-week/three-month loadings (PC1-PC6).	16](#_Toc225867000)

[**Supplementary Figure 5**: Univariate associations between maternal and child predictors and top six principal components (PCs) at three weeks postpartum. The heatmap displays beta-coefficients from linear regression models, with red indicating a positive association and blue, negative. The intensity of the colour corresponds to the strength of the associations, while asterisks denote statistical significance (* p<0.05, **p<0.01, *** p<0.001). Abbreviations: PC, principal component; BW, birth weight; SGA, small for gestational age; AGA, appropriate for gestational age; LGA, large for gestational age; CS, caesarean section; BF, breastfeeding; GWG, gestational weight gain; PA, physical activity; ppBMI, pre-pregnancy body mass index; STAI-S, State-Trait Anxiety Inventory-State; STAI-T, State-Trait Anxiety Inventory-Trait.	17](#_Toc225867001)

[**Supplementary Figure 6**: Multivariate associations between selected significant predictors (from univariate model) and top six principal components (PC) at three weeks postpartum. The heatmap displays beta coefficients from a multivariate regression, with red and blue demonstrating positive and negative associations, respectively. Colour intensities demonstrate strength of associations, while p-values are represented by asterisks denoting statistical significance (* p adj<0.05, ** p adj<0.01). The bar plot (left) displays adjusted R2 values for each principal component; the selected predictors explain 22%,10%, 24%, 4%, 7% and 7% of the variances of PCs 1-6.	18](#_Toc225867002)

[**Supplementary Figure 7**: Stacked bar-chart illustrating the relative contribution (% R2) of individual factors, such as ethnicity, breastfeeding, antenatal maternal anxiety (STAI-S), birthweight and maternal education on each principal component (PC1-PC6) at three weeks	19](#_Toc225867003)

[**Supplementary Figure 8**: Univariate associations between maternal and child predictors and top six principal components (PCs) at three months postpartum. The heatmap displays beta-coefficients from linear regression models, with red indicating a positive association and blue, negative. The intensity of the colour corresponds to the strength of the associations, while asterisks denote statistical significance (* p<0.05, **p<0.01, *** p<0.001). Abbreviations: PC, principal component; BW, birth weight; SGA, small for gestational age; AGA, appropriate for gestational age; LGA, large for gestational age; CS, caesarean section; BF, breastfeeding; GWG, gestational weight gain; PA, physical activity; ppBMI, pre-pregnancy body mass index; STAI-S, State-Trait Anxiety Inventory-State; STAI-T, State-Trait Anxiety Inventory-Trait.	21](#_Toc225867004)

[**Supplementary Figure 9**: Multivariate associations between selected significant predictors (from univariate model) and top six principal components (PC) at three months postpartum. The heatmap displays beta coefficients from a multivariate regression, with red and blue demonstrating positive and negative associations, respectively. Colour intensities demonstrate strength of associations, while p-values are represented by asterisks denoting statistical significance (* p adj<0.05, ** p adj<0.01). The bar plot (left) displays adjusted R^2^ values for each principal component; the selected predictors explain 19%,30%, 20%, 1%, 4% and 23% of the variances of PCs 1-6.	22](#_Toc225867005)

[**Supplementary Figure 10**: Stacked bar-chart illustrating the relative contribution (% R2) of individual factors, such as ethnicity, breastfeeding, maternal age, maternal education and tobacco smoke exposure on each principal component (PC1-PC6) at three months	23](#_Toc225867006)

[**Supplementary Figure 11**: Maternal fish oil supplementation by ethnicity at three weeks postpartum. P-values determined by Fisher’s exact test	24](#_Toc225867007)

[**Supplementary Figure 12**: Linkage disequilibrium (r²) heatmap comparing 15 SNPs in the FADS1/FADS2/FADS3 gene cluster.	24](#_Toc225867008)

[**Supplementary Table 1**: Internal standard concentrations (nM) in final measured sample	6](#_Toc225427412)

[**Supplementary Table 2**: Full participant characteristics by Timepoint	7](#_Toc225427413)

[**Supplementary Table 3**: Ethnic differences in 18 SNPs from FADS1/2/3 gene cluster. Table reports SNP annotations (chromosome, position, strand, genotype sequence), reference/alternate allele in GUSTO cohort, unadjusted and adjusted global p values from ethnic comparisons.	11](#_Toc225427414)

[**Supplementary Table** **4**: SNP annotations and alternate allele frequencies (n = 180)	12](#_Toc225427415)

[**Supplementary Table 5 :** Pairwise genotype comparisons by ethnicity with Pearson’s chi squared or (Fisher’s exact test used when n<5).	12](#_Toc225427416)

[**Supplementary Table 6** : Assessment of multicollinearity amongst predictor variables included in multivariate analysis at three weeks using adjusted variance inflation factors (VIF).	20](#_Toc225427417)

[**Supplementary Table 7**: Assessment of multicollinearity amongst predictor variables included in multivariate analysis at three months using adjusted variance inflation factors (VIF).	23](#_Toc225427418)

[**Supplementary Table 8** : Full MRM transition list used for GUSTO cohort (PL and SP only) – before lipid inclusion criteria	25](#_Toc225427419)

**Supplementary Figure 1 :** Flowchart detailing the exclusion and inclusion criteria for the selection of human milk samples from the GUSTO (Growing Up in Singapore Towards healthy Outcomes) cohort leading to the final sample numbers used at the 3-week and 3-month postpartum timepoints.

**Lipid quantification and Data processing**

The peak areas/intensities of the endogenous lipids were normalised to the areas of the internal standards (specific for each class). The only exceptions were DGs, which were normalised to the TG 48: 0 d5 internal standard and Hex2Cers, which were normalised to Hex1Cer d18:1/08:0. Relative concentrations were calculated using the following formula:

C_cmpd = (PA_Cmpd / PA_IS) × C_IS × Dilution Factor

Where,

C = concentration,

PA= Peak Area,

Cmpd = Compound,

ISTD = Internal Standard,

Dilution Factor: 20 for PL and SP; 200 for TG and DG

Batch correction using the locally weighted scatter-plot smoother (LOESS) was applied for the following lipids : Cer d18:1/23:0; Hex2Cer d18:1/16:0; LPC 15:0; LPC 16:0; LPC 18:0; LPC 18:1; LPC 18:2, LPC 18:3; LPC 20:1; LPC 20:2; LPC 20:3; LPC 20:4; LPC 20:5; LPC 22:6; LPE 18:0; LPE 18:1; LPE 20:1; LPE 20:4; LPE 22:1; LPE 22:5; LPE 22:6; SM 40:0; SM 41:1; SM 42:0; SM 43:1; PC 38:3. No batch effect was observed for any of the other lipids.

Missing values were replaced by using half of the minimum of non-missing values for each lipid (1).

A secondary normalisation known as the probabilistic quotient normalisation (PQN)(2) was performed for the GUSTO samples.

**Supplementary Table 1**: Internal standard concentrations (nM) in final measured sample

| Internal Standard (ISTD) | Concentration of ISTD in 200 μL - for PL & SP analysis (nM) | Concentration of ISTD diluted 10 X (nM) for TG |
| --- | --- | --- |
| Glyceryl trihexadecanoate (TG 48:0) d5 | nil | 500 |
| Lysophosphatidylethanolamine (LPE) 14:0 | 200 | nil |
| Lysophosphatidylcholine (LPC) 13:0 | 250 | nil |
| Phosphatidylcholine (PC) 13:0_13:0 | 250 | nil |
| Phosphatidylethanolamine 14:0_14:0 | 200 | nil |
| Phosphatidylserine (PS) 14:0_14:0 | 500 | nil |
| Sphingomyelin (SM) d18:1/06:0 | 350 | nil |
| Glucosyl C8 Ceramide (Hex1Cer) d18:1/08:0 | 40 | nil |
| Ceramide (Cer) d18:1/17:0 | 25 | nil |
| Phosphatidylinositol (PI) 12:0_13:0 | 400 | nil |

**Supplementary Table 2**: Full participant characteristics by Timepoint

|  | **Summary by Timepoint** | |
| --- | --- | --- |
| **Variable** | **3 Weeks**  N = 189^1^ | **3 Months**  N = 104^1^ |
| **Maternal Age** |  |  |
| 18-26 | 44 (23%) | 19 (18%) |
| 27-34 | 112 (59%) | 61 (59%) |
| ≥35 | 33 (17%) | 24 (23%) |
| **Ethnicity** |  |  |
| Chinese | 137 (72%) | 84 (81%) |
| Malay | 29 (15%) | 10 (9.6%) |
| Indian | 23 (12%) | 10 (9.6%) |
| **Maternal Education (Highest)** |  |  |
| University education | 95 (50%) | 63 (61%) |
| Non-university education | 94 (50%) | 40 (39%) |
| NA | 0 | 1 |
| **Pre-pregnancy BMI (Asian, kg/m²)** |  |  |
| Normal (18.5-22.9) | 100 (55%) | 60 (63%) |
| Underweight (<18.5) | 20 (11%) | 11 (12%) |
| Overweight/Obese (≥23) | 61 (34%) | 24 (25%) |
| NA | 8 | 9 |
| **Parity** |  |  |
| Nulliparous | 91 (48%) | 52 (50%) |
| Parous | 98 (52%) | 52 (50%) |
| **Tobacco Smoke Exposure** |  |  |
| No exposure | 115 (64%) | 71 (74%) |
| Self-reported exposure (no detectable plasma cotinine) | 46 (26%) | 20 (21%) |
| Biomarker plasma cotinine-confirmed exposure | 18 (10%) | 5 (5.2%) |
| NA | 10 | 8 |
| **Physical Activity Before Pregnancy (MET/week)^a^** |  |  |
| <600 | 38 (20%) | 23 (23%) |
| 600-3000 | 105 (56%) | 57 (56%) |
| 3000+ | 45 (24%) | 21 (21%) |
| NA | 1 | 3 |
| **Physical Activity During Pregnancy (MET/week)** |  |  |
| <600 | 65 (35%) | 46 (45%) |
| 600-3000 | 93 (49%) | 38 (37%) |
| 3000+ | 30 (16%) | 18 (18%) |
| NA | 1 | 2 |
| **STAI-S at 26 weeks** |  |  |
| STAI-S < 41 | 146 (81%) | 81 (83%) |
| STAI-S ≥ 41 | 35 (19%) | 17 (17%) |
| NA | 8 | 6 |
| **STAI-T at 26 weeks** |  |  |
| STAI-T < 41 | 139 (77%) | 72 (73%) |
| STAI-T ≥ 41 | 42 (23%) | 26 (27%) |
| NA | 8 | 6 |
| **Gestational Weight Gain (IOM)^b^** |  |  |
| Excessive | 67 (38%) | 30 (32%) |
| Normal | 68 (38%) | 43 (45%) |
| Inadequate | 43 (24%) | 22 (23%) |
| NA | 11 | 9 |
| **Mode of Delivery** |  |  |
| Non-labour C-section | 22 (12%) | 12 (12%) |
| Intrapartum C-section | 24 (13%) | 12 (12%) |
| Vaginal | 143 (76%) | 80 (77%) |
| **Gestational Age at Birth (weeks)** | 39.09 (0.98) | 39.16 (0.96) |
| **Infant Sex** |  |  |
| Female | 94 (50%) | 48 (46%) |
| Male | 95 (50%) | 56 (54%) |
| **Birth Size/Weight^c^** |  |  |
| Appropriate for Gestational Age (AGA) | 138 (73%) | 79 (76%) |
| Small for Gestational Age (SGA) | 17 (9.0%) | 4 (3.8%) |
| Large for Gestational Age (LGA) | 34 (18%) | 21 (20%) |
| **Exclusive/Predominant ^d^ BF (3 Weeks)** |  |  |
| Not | 124 (66%) | - |
| Yes | 63 (34%) | - |
| NA | 2 | - |
| **Exclusive/Predominant BF (3 Months)** |  |  |
| Not | - | 57 (55%) |
| Yes | - | 47 (45%) |
| NA | - | 0 |
| ^1^Mean (SD); n (%) ᵃ Physical activity measured in metabolic equivalent task (MET) per week as defined by WHO (2010)^(3)^. ᵇ Gestational weight gain defined as recommended by the Institute of Medicine (2009)^(4)^. ᶜ Birth size defined according to Mikolajczyk (2011)^(5)^. ᵈ Exclusive or Predominant Breastfeeding defined as no non-human milk or solids; may have consumed water, fruit juices, drops, and syrups (vitamins and minerals) as published in Pang (2016)^(6)^. Abbreviations: BMI, Body Mass Index; BF, Breastfeeding; C-section, Caesarean section; STAI-S/T, State–Trait Anxiety Inventory (State/Trait). | | |

**Linkage disequilibrium patterns**

We examined whether genetic factors, particularly 15 SNPs from the FADS1, FADS2 and FADS3 variants may have contributed to the observed ethnic differences. Among the 18 SNPs that passed QC, 15 showed significant differences by ethnicity (p adj <0.05, Supplementary Table 3). Analysis of linkage disequilibrium (LD) patterns (Supplementary Figure 12) revealed four distinct groups that capture different aspects of genetic variation across the FADS gene cluster. The first group comprised most of the FADS1 and FADS2 variants, with the FADS1 SNPs, rs174546, rs174547, rs174548, rs174550, and FADS2 SNPs, rs174570, rs1535, rs174576, rs174577, and rs174583 displaying very high correlation (r^2^ =0.97-1), allowing us to select one representative SNP, rs174583. The second group consisted of the FADS2 variants, rs2727270 and rs2851682, which had moderate correlation with the first group (r^2^= 0.61-0.65), but high correlation with each other (r^2^ = 0.93). The third group consisted of a single, independent SNP from FADS2, rs498793, which did not correlate with any of the other groups (r^2^ = 0.002-0.03). Finally, the last group included the FADS3 SNPs, rs174448, rs174449 and rs174450, which showed low correlation with all three aforementioned groups (r^2^= 0.05-0.27), but displayed moderate to high correlations between themselves (r^2^=0.45-0.71). These distinct LD patterns suggest that there might be multiple independent genetic associations with the milk lipids, which may help to explain ethnic-based differences. For this paper, we selected rs174583 and rs2727270 as representative SNPs.

**Supplementary Table 3**: Ethnic differences in 18 SNPs from FADS1/2/3 gene cluster. Table reports SNP annotations (chromosome, position, strand, genotype sequence), reference/alternate allele in GUSTO cohort, unadjusted and adjusted global p values from ethnic comparisons.

| **SNP** | **FADS Gene** | **Ref/Alt^b^** | **Global_p** | **Global method^a^** | **Global_p_BH** |
| --- | --- | --- | --- | --- | --- |
| rs174546 | FADS1 | A/G | 6.53E-10 | chisq | 1.93E-09 |
| rs174547 | FADS1 | G/A | 6.53E-10 | chisq | 1.93E-09 |
| rs174548 | FADS1 | C/G | 1.00E-09 | chisq | 2.25E-09 |
| rs174550 | FADS1 | G/A | 6.53E-10 | chisq | 1.93E-09 |
| rs174570 | FADS2 | A/G | 6.92E-12 | chisq | 1.25E-10 |
| rs1535 | FADS2 | G/A | 5.49E-11 | chisq | 4.94E-10 |
| rs2727270 | FADS2 | G/A | 1.37E-08 | fisher | 2.47E-08 |
| rs174576 | FADS2 | A/C | 7.52E-10 | chisq | 1.93E-09 |
| rs174577 | FADS2 | A/C | 7.52E-10 | chisq | 1.93E-09 |
| rs174583 | FADS2 | A/G | 8.29E-09 | chisq | 1.66E-08 |
| rs2851682 | FADS2 | A/G | 2.49E-08 | fisher | 4.08E-08 |
| rs174593 | FADS2 | A/G | 0.0717 | fisher | 0.0806 |
| rs498793 | FADS2 | G/A | 6.31E-07 | fisher | 9.47E-07 |
| rs174448 | FADS3 | A/G | 0.00741 | fisher | 0.0103 |
| rs174449 | FADS3 | A/G | 0.0086 | fisher | 0.0111 |
| rs174450 | FADS3 | A/C | 0.0132 | fisher | 0.0158 |
| rs1000778 | FADS3 | G/A | 0.392 | fisher | 0.392 |
| rs174455 | FADS3 | A/G | 0.0823 | fisher | 0.0871 |

^a^ Ethnic comparisons performed using chi-square test; Fisher’s exact test used when counts < 5.

**Supplementary Table** **4**: SNP annotations and alternate allele frequencies (n = 180)

**^a^** Forward or reverse strand denoted by +/-

| **SNP** | **FADS_Gene** | **Chr** | **Position** | **Strand^a^** | **Ref/Alt** | **Alternate allele freq** |  |
| --- | --- | --- | --- | --- | --- | --- | --- |
| **rs174546** | FADS1 | 11 | 61569830 | - | A/G | 0.46 |  |
| **rs174547** | FADS1 | 11 | 61570783 | - | G/A | 0.46 |  |
| **rs174548** | FADS1 | 11 | 61571348 | - | C/G | 0.46 |  |
| **rs174550** | FADS1 | 11 | 61571478 | - | G/A | 0.46 |  |
| **rs174570** | FADS2 | 11 | 61597212 | - | A/G | 0.47 |  |
| **rs1535** | FADS2 | 11 | 61597972 | + | G/A | 0.46 |  |
| **rs2727270** | FADS2 | 11 | 61603237 | - | G/A | 0.42 |  |
| **rs174576** | FADS2 | 11 | 61603510 | + | A/C | 0.45 |  |
| **rs174577** | FADS2 | 11 | 61604814 | + | A/C | 0.45 |  |
| **rs174583** | FADS2 | 11 | 61609750 | - | A/G | 0.45 |  |
| **rs2851682** | FADS2 | 11 | 61616012 | + | A/G | 0.41 |  |
| **rs498793** | FADS2 | 11 | 61624705 | - | G/A | 0.13 |  |
| **rs174448** | FADS3 | 11 | 61639573 | + | A/G | 0.32 |  |
| **rs174449** | FADS3 | 11 | 61640379 | + | A/G | 0.37 |  |
| **rs174450** | FADS3 | 11 | 61641542 | - | A/C | 0.44 |  |

**Supplementary Table 5 :** Pairwise genotype comparisons by ethnicity with Pearson’s chi squared or (Fisher’s exact test used when n<5).

| SNP | Ethnicity | Ethnicity | Pairwise  method | Pairwise p BH corrected |
| --- | --- | --- | --- | --- |
| rs174546 | Chinese | Malay | fisher | 0.0939 |
| rs174546 | Chinese | Indian | chisq | **3.02E-08** |
| rs174546 | Malay | Indian | chisq | **1.96E-07** |
| rs174547 | Chinese | Malay | fisher | 0.0939 |
| rs174547 | Chinese | Indian | chisq | **3.02E-08** |
| rs174547 | Malay | Indian | chisq | **1.96E-07** |
| rs174548 | Chinese | Malay | fisher | 0.132 |
| rs174548 | Chinese | Indian | chisq | **3.07E-08** |
| rs174548 | Malay | Indian | chisq | **2.24E-07** |
| rs174550 | Chinese | Malay | fisher | 0.0939 |
| rs174550 | Chinese | Indian | chisq | **3.02E-08** |
| rs174550 | Malay | Indian | chisq | **1.96E-07** |
| rs174570 | Chinese | Malay | chisq | 0.0659 |
| rs174570 | Chinese | Indian | chisq | **6.08E-10** |
| rs174570 | Malay | Indian | chisq | **1.62E-08** |
| rs1535 | Chinese | Malay | fisher | 0.0939 |
| rs1535 | Chinese | Indian | chisq | **3.18E-09** |
| rs1535 | Malay | Indian | chisq | **6.17E-08** |
| rs2727270 | Chinese | Malay | chisq | 0.39 |
| rs2727270 | Chinese | Indian | fisher | **2.27E-08** |
| rs2727270 | Malay | Indian | fisher | **3.63E-08** |
| rs174576 | Chinese | Malay | fisher | 0.114 |
| rs174576 | Chinese | Indian | chisq | **2.98E-08** |
| rs174576 | Malay | Indian | chisq | **1.96E-07** |
| rs174577 | Chinese | Malay | fisher | 0.114 |
| rs174577 | Chinese | Indian | chisq | **2.98E-08** |
| rs174577 | Malay | Indian | chisq | **1.96E-07** |
| rs174583 | Chinese | Malay | fisher | 0.114 |
| rs174583 | Chinese | Indian | chisq | **2.30E-07** |
| rs174583 | Malay | Indian | chisq | **5.36E-07** |
| rs2851682 | Chinese | Malay | chisq | 0.326 |
| rs2851682 | Chinese | Indian | fisher | **3.63E-08** |
| rs2851682 | Malay | Indian | fisher | **3.63E-08** |
| rs498793 | Chinese | Malay | fisher | **0.0479** |
| rs498793 | Chinese | Indian | fisher | **1.14E-06** |
| rs498793 | Malay | Indian | fisher | **0.00597** |
| rs174448 | Chinese | Malay | fisher | **0.00315** |
| rs174448 | Chinese | Indian | fisher | 0.941 |
| rs174448 | Malay | Indian | fisher | 0.116 |
| rs174449 | Chinese | Malay | fisher | **0.00631** |
| rs174449 | Chinese | Indian | fisher | 0.676 |
| rs174449 | Malay | Indian | chisq | 0.0967 |
| rs174450 | Chinese | Malay | chisq | **0.0132** |
| rs174450 | Chinese | Indian | fisher | 0.351 |
| rs174450 | Malay | Indian | chisq | **0.0132** |

**Supplementary Figure 2**: Scree plot shows explained variance (%) of each principal component derived from PCA of lipidomic dataset (both three weeks and three months)

**Supplementary Figure 3**: Simplified path diagrams shows the top positive and negative loadings of lipid species driving variation in PCs 4-6: (A) PC4 (8.8% of variation), (B) PC5 (6.2%), and (C) PC6 (4.4%). Lipids are classified into specific categories with exact loading values displayed beside each species. Red and blue indicate positive and negative associations, respectively; the width of each line represents median absolute loading of the most strongly associated lipids within that classification. Abbreviations: PL, phospholipids; PC, phosphatidylcholine; PE, phosphatidylethanolamine LPC, lysophosphatidylcholine; LPE, lysophosphatidylethanolamine; PS, phosphatidylserine; PI, phosphatidylinositol; SM, sphingomyelin; DAG, diacylglycerol; TG, triacylglycerol; Cer, ceramide; Hex1Cer, monohexosylceramide; Hex2Cer, dihexosylceramide. MUFA, monounsaturated fatty acids; PUFA, polyunsaturated fatty acids; n-3, omega-3 fatty acids; DB, double bonds.

**Concordance analysis of combined and timepoint-specific PCAs**

We compared principal components by correlating the loading vectors derived from the combined (3W and 3M) dataset to those derived from the individual three-week (3W) and three-month (3M) datasets. Combined PC1 and PC2 showed strong correlations (Pearson’s r) with 3W PC1 and PC2 (r = 0.84 and r = 0.93, respectively). Subsequent combined PCs showed moderate correlations with 3W data (r = 0.59 and r = 0.48, respectively). Several notable off-diagonal matches were also observed (combined PC4 with 3W PC3, r = 0.67; combined PC5 with 3W PC4, r = 0.81), indicating a reordering of the axes. For the 3M comparison, the strongest correlation was off-diagonal (combined PC4 with 3M PC3, r = 0.96) with additional moderate correlations across diagonals ( combined PC1 with 3M PC1, r = -0.61; combined PC2 with 3M PC2, r = 0.68) and off-diagonals (combined PC1 with 3M PC2, r = 0.65; combined PC2 with 3M PC1, r = 0.68). Similarity between the combined data and the timepoint specific 3W and 3M loadings (PC1-PC6) was assessed using Escoufier’s RV coefficient and shows near-identical similarity (RV = 0.994 and RV =0.999, respectively), supporting that the combined PCA captures the same underlying lipid patterns compared to the time-specific PCAs (Supplementary Figure 4).

|  | 3w_PC1 | 3w_PC2 | 3w_PC3 | 3w_PC4 | 3w_PC5 | 3w_PC6 |
| --- | --- | --- | --- | --- | --- | --- |
| Combined_PC1 | 0.84 | -0.27 | -0.21 | 0.04 | -0.23 | -0.03 |
| Combined_PC2 | 0.35 | 0.93 | 0.08 | -0.02 | 0.03 | 0.02 |
| Combined_PC3 | 0.39 | -0.24 | 0.59 | -0.11 | 0.42 | -0.01 |
| Combined_PC4 | -0.08 | 0.00 | 0.67 | 0.48 | -0.50 | -0.04 |
| Combined_PC5 | 0.03 | 0.02 | -0.21 | 0.81 | 0.50 | -0.10 |
| Combined_PC6 | -0.06 | 0.03 | 0.31 | -0.25 | 0.44 | 0.10 |
|  | 3m_PC1 | 3m_PC2 | 3m_PC3 | 3m_PC4 | 3m_PC5 | 3m_PC6 |
| Combined_PC1 | -0.61 | 0.65 | -0.08 | -0.19 | 0.07 | 0.17 |
| Combined_PC2 | 0.68 | 0.68 | -0.15 | 0.12 | 0.05 | -0.05 |
| Combined_PC3 | -0.32 | 0.24 | 0.11 | 0.44 | -0.38 | -0.33 |
| Combined_PC4 | 0.09 | 0.12 | 0.96 | 0.01 | -0.01 | 0.06 |
| Combined_PC5 | -0.10 | -0.06 | -0.01 | 0.60 | 0.62 | 0.35 |
| Combined_PC6 | 0.01 | -0.07 | -0.10 | 0.58 | -0.39 | -0.03 |
| Comparison | RV Coefficient | | | | | |
| Combined_vs_3w | 0.994 | | | | | |
| Combined_vs_3m | 0.999 | | | | | |

**Supplementary Figure 4**: Concordance analysis using Pearson’s correlations between vector loadings from combined PCA and three-week (top) and three-month (bottom) datasets. Escoufier’s RV coefficients are displayed to show global subspace similarity between the combined data and the three-week/three-month loadings (PC1-PC6).

**Supplementary Figure 5**: Univariate associations between maternal and child predictors and top six principal components (PCs) at three weeks postpartum. The heatmap displays beta-coefficients from linear regression models, with red indicating a positive association and blue, negative. The intensity of the colour corresponds to the strength of the associations, while asterisks denote statistical significance (* p<0.05, **p<0.01, *** p<0.001). Abbreviations: PC, principal component; BW, birth weight; SGA, small for gestational age; AGA, appropriate for gestational age; LGA, large for gestational age; CS, caesarean section; BF, breastfeeding; GWG, gestational weight gain; PA, physical activity; ppBMI, pre-pregnancy body mass index; STAI-S, State-Trait Anxiety Inventory-State; STAI-T, State-Trait Anxiety Inventory-Trait.

**Supplementary Figure 6**: Multivariate associations between selected significant predictors (from univariate model) and top six principal components (PC) at three weeks postpartum. The heatmap displays beta coefficients from a multivariate regression, with red and blue demonstrating positive and negative associations, respectively. Colour intensities demonstrate strength of associations, while p-values are represented by asterisks denoting statistical significance (* p adj<0.05, ** p adj<0.01). The bar plot (left) displays adjusted R2 values for each principal component; the selected predictors explain 22%,10%, 24%, 4%, 7% and 7% of the variances of PCs 1-6.

**Supplementary Figure 7**: Stacked bar-chart illustrating the relative contribution (% R2) of individual factors, such as ethnicity, breastfeeding, antenatal maternal anxiety (STAI-S), birthweight and maternal education on each principal component (PC1-PC6) at three weeks

**Supplementary Table 6** : Assessment of multicollinearity amongst predictor variables included in multivariate analysis at three weeks using adjusted variance inflation factors (VIF).

**Supplementary Figure 8**: Univariate associations between maternal and child predictors and top six principal components (PCs) at three months postpartum. The heatmap displays beta-coefficients from linear regression models, with red indicating a positive association and blue, negative. The intensity of the colour corresponds to the strength of the associations, while asterisks denote statistical significance (* p<0.05, **p<0.01, *** p<0.001). Abbreviations: PC, principal component; BW, birth weight; SGA, small for gestational age; AGA, appropriate for gestational age; LGA, large for gestational age; CS, caesarean section; BF, breastfeeding; GWG, gestational weight gain; PA, physical activity; ppBMI, pre-pregnancy body mass index; STAI-S, State-Trait Anxiety Inventory-State; STAI-T, State-Trait Anxiety Inventory-Trait.

**Supplementary Figure 9**: Multivariate associations between selected significant predictors (from univariate model) and top six principal components (PC) at three months postpartum. The heatmap displays beta coefficients from a multivariate regression, with red and blue demonstrating positive and negative associations, respectively. Colour intensities demonstrate strength of associations, while p-values are represented by asterisks denoting statistical significance (* p adj<0.05, ** p adj<0.01). The bar plot (left) displays adjusted R^2^ values for each principal component; the selected predictors explain 19%,30%, 20%, 1%, 4% and 23% of the variances of PCs 1-6.

**Supplementary Figure 10**: Stacked bar-chart illustrating the relative contribution (% R2) of individual factors, such as ethnicity, breastfeeding, maternal age, maternal education and tobacco smoke exposure on each principal component (PC1-PC6) at three months

**Supplementary Table 7**: Assessment of multicollinearity amongst predictor variables included in multivariate analysis at three months using adjusted variance inflation factors (VIF).

**Supplementary Figure 11**: Maternal fish oil supplementation by ethnicity at three weeks postpartum. P-values determined by Fisher’s exact test

**Supplementary Figure 12**: Linkage disequilibrium (r²) heatmap comparing 15 SNPs in the FADS1/FADS2/FADS3 gene cluster.

**Supplementary Table 8** : Full MRM transition list used for GUSTO cohort (PL and SP only) – before lipid inclusion criteria

| Cpd Group | Cpd Name | Prec Ion | Prod Ion | Polarity |
| --- | --- | --- | --- | --- |
| Cer | Cer d16:1/16:0 | 510.5 | 236.4 | Positive |
| Cer | Cer d16:1/18:0 | 538.5 | 236.4 | Positive |
| Cer | Cer d16:1/20:0 | 566.5 | 236.4 | Positive |
| Cer | Cer d16:1/22:0 | 594.7 | 236.4 | Positive |
| Cer | Cer d16:1/23:0 | 608.7 | 236.4 | Positive |
| Cer | Cer d16:1/24:0 | 622.7 | 236.4 | Positive |
| Cer | Cer d16:1/24:1 | 620.7 | 236.4 | Positive |
| Cer | Cer d17:0/25:2 | 648.6 | 252.3 | Positive |
| Cer | Cer d17:1/16:0 | 524.6 | 250.4 | Positive |
| Cer | Cer d17:1/18:0 | 552.6 | 250.4 | Positive |
| Cer | Cer d17:1/20:0 | 580.6 | 250.4 | Positive |
| Cer | Cer d17:1/22:0 | 608.6 | 250.4 | Positive |
| Cer | Cer d17:1/23:0 | 622.6 | 250.4 | Positive |
| Cer | Cer d17:1/24:0 | 636.6 | 250.4 | Positive |
| Cer | Cer d17:1/24:1 | 634.6 | 250.4 | Positive |
| dhCer | Cer d18:0/08:0 | 428.4 | 284.3 | Positive |
| dhCer | Cer d18:0/16:0 | 540.5 | 284.3 | Positive |
| dhCer | Cer d18:0/18:0 | 568.6 | 284.3 | Positive |
| dhCer | Cer d18:0/20:0 | 596.6 | 284.3 | Positive |
| dhCer | Cer d18:0/22:0 | 624.6 | 284.3 | Positive |
| dhCer | Cer d18:0/23:0 | 638.6 | 284.3 | Positive |
| dhCer | Cer d18:0/24:0 | 652.7 | 284.3 | Positive |
| dhCer | Cer d18:0/24:1 | 650.6 | 284.3 | Positive |
| Cer | Cer d18:1/16:0 | 538.5 | 264.3 | Positive |
| Cer | Cer d18:1/17:0 (IS) | 552.5 | 264.3 | Positive |
| Cer | Cer d18:1/18:0 | 566.6 | 264.3 | Positive |
| Cer | Cer d18:1/19:0 | 580.5 | 264.3 | Positive |
| Cer | Cer d18:1/20:0 | 594.6 | 264.3 | Positive |
| Cer | Cer d18:1/21:0 | 608.6 | 264.3 | Positive |
| Cer | Cer d18:1/22:0 | 622.6 | 264.3 | Positive |
| Cer | Cer d18:1/23:0 | 636.6 | 264.3 | Positive |
| Cer | Cer d18:1/24:0 | 650.6 | 264.3 | Positive |
| Cer | Cer d18:1/24:1 | 648.6 | 264.3 | Positive |
| Cer | Cer d18:1/25:0 | 664.7 | 264.3 | Positive |
| Cer | Cer d18:1/26:1 | 676.7 | 264.3 | Positive |
| Cer | Cer d18:2/16:0 | 536.5 | 262.3 | Positive |
| Cer | Cer d18:2/17:0 | 550.5 | 262.3 | Positive |
| Cer | Cer d18:2/18:0 | 564.6 | 262.3 | Positive |
| Cer | Cer d18:2/19:0 | 578.6 | 262.3 | Positive |
| Cer | Cer d18:2/20:0 | 592.6 | 262.3 | Positive |
| Cer | Cer d18:2/21:0 | 606.6 | 262.3 | Positive |
| Cer | Cer d18:2/22:0 | 620.6 | 262.3 | Positive |
| Cer | Cer d18:2/23:0 | 634.6 | 262.3 | Positive |
| Cer | Cer d18:2/24:0 | 648.6 | 262.3 | Positive |
| Cer | Cer d18:2/24:1 | 646.6 | 262.3 | Positive |
| Cer | Cer d18:2/26:1 | 674.6 | 262.3 | Positive |
| Cer | Cer d19:1/16:0 | 552.6 | 278.3 | Positive |
| Cer | Cer d19:1/18:0 | 580.6 | 278.3 | Positive |
| Cer | Cer d19:1/20:0 | 608.6 | 278.3 | Positive |
| Cer | Cer d19:1/22:0 | 636.6 | 278.3 | Positive |
| Cer | Cer d19:1/23:0 | 650.6 | 278.3 | Positive |
| Cer | Cer d19:1/24:0 | 664.6 | 278.3 | Positive |
| Cer | Cer d19:1/24:1 | 662.6 | 278.3 | Positive |
| Cer | Cer d20:1/16:0 | 566.6 | 292.3 | Positive |
| Cer | Cer d20:1/18:0 | 594.6 | 292.3 | Positive |
| Cer | Cer d20:1/20:0 | 622.6 | 292.3 | Positive |
| Cer | Cer d20:1/22:0 | 650.6 | 292.3 | Positive |
| Cer | Cer d20:1/23:0 | 664.6 | 292.3 | Positive |
| Cer | Cer d20:1/24:0 | 678.6 | 292.3 | Positive |
| Cer | Cer d20:1/24:1 | 676.6 | 292.3 | Positive |
| GM3 | GM3 d18:1/16:0 | 1153.7 | 264.3 | Positive |
| GM3 | GM3 d18:1/18:0 | 1181.8 | 264.3 | Positive |
| GM3 | GM3 d18:1/18:0 d3 (IS) | 1184.8 | 264.3 | Positive |
| GM3 | GM3 d18:1/20:0 | 1209.8 | 264.3 | Positive |
| GM3 | GM3 d18:1/22:0 | 1237.8 | 264.3 | Positive |
| GM3 | GM3 d18:1/24:0 | 1265.8 | 264.3 | Positive |
| GM3 | GM3 d18:1/24:1 | 1263.8 | 264.3 | Positive |
| Hex1Cer | Hex1Cer d16:1/18:0 | 700.6 | 236.3 | Positive |
| Hex1Cer | Hex1Cer d16:1/20:0 | 728.6 | 236.3 | Positive |
| Hex1Cer | Hex1Cer d16:1/22:0 | 756.7 | 236.3 | Positive |
| Hex1Cer | Hex1Cer d16:1/24:0 | 784.7 | 236.3 | Positive |
| Hex1Cer | Hex1Cer d18:1/08:0 (IS) | 588.8 | 264.3 | Positive |
| Hex1Cer | Hex1Cer d18:1/16:0 | 700.6 | 264.3 | Positive |
| Hex1Cer | Hex1Cer d18:1/18:0 | 728.6 | 264.3 | Positive |
| Hex1Cer | Hex1Cer d18:1/20:0 | 756.6 | 264.3 | Positive |
| Hex1Cer | Hex1Cer d18:1/22:0 | 784.7 | 264.3 | Positive |
| Hex1Cer | Hex1Cer d18:1/24:0 | 812.7 | 264.3 | Positive |
| Hex1Cer | Hex1Cer d18:1/24:1 | 810.7 | 264.3 | Positive |
| Hex1Cer | Hex1Cer d18:2/18:0 | 726.6 | 262.3 | Positive |
| Hex1Cer | Hex1Cer d18:2/20:0 | 754.6 | 262.3 | Positive |
| Hex1Cer | Hex1Cer d18:2/22:0 | 782.7 | 262.3 | Positive |
| Hex1Cer | Hex1Cer d18:2/24:0 | 810.7 | 262.3 | Positive |
| Hex2Cer | Hex2Cer d16:1/18:0 | 862.6 | 236.3 | Positive |
| Hex2Cer | Hex2Cer d16:1/20:0 | 890.7 | 236.3 | Positive |
| Hex2Cer | Hex2Cer d16:1/22:0 | 918.7 | 236.3 | Positive |
| Hex2Cer | Hex2Cer d16:1/24:0 | 946.7 | 236.3 | Positive |
| Hex2Cer | Hex2Cer d18:1/16:0 | 862.6 | 264.3 | Positive |
| Hex2Cer | Hex2Cer d18:1/20:0 | 918.7 | 264.3 | Positive |
| Hex2Cer | Hex2Cer d18:1/22:0 | 946.7 | 264.3 | Positive |
| Hex2Cer | Hex2Cer d18:1/24:0 | 974.8 | 264.3 | Positive |
| Hex2Cer | Hex2Cer d18:1/24:1 | 972.7 | 264.3 | Positive |
| Hex2Cer | Hex2Cer d18:2/18:0 | 888.7 | 262.3 | Positive |
| Hex2Cer | Hex2Cer d18:2/20:0 | 916.7 | 262.3 | Positive |
| Hex2Cer | Hex2Cer d18:2/22:0 | 944.7 | 262.3 | Positive |
| Hex2Cer | Hex2Cer d18:2/24:0 | 972.7 | 262.3 | Positive |
| Hex3Cer | Hex3Cer d18:1/16:0 | 1024.7 | 264.3 | Positive |
| Hex3Cer | Hex3Cer d18:1/18:0 | 1052.7 | 264.3 | Positive |
| Hex3Cer | Hex3Cer d18:1/20:0 | 1080.7 | 264.3 | Positive |
| Hex3Cer | Hex3Cer d18:1/22:0 | 1108.8 | 264.3 | Positive |
| Hex3Cer | Hex3Cer d18:1/24:0 | 1136.8 | 264.3 | Positive |
| Hex3Cer | Hex3Cer d18:1/24:1 | 1134.8 | 264.3 | Positive |
| LPC | LPC 13:0 (IS) | 454.3 | 184.1 | Positive |
| LPC | LPC 14:0 | 468.3 | 184.1 | Positive |
| LPC | LPC 15:0 | 482.3 | 184.1 | Positive |
| LPC | LPC 16:0 | 496.3 | 184.1 | Positive |
| LPC | LPC 16:1 | 494.3 | 184.1 | Positive |
| LPC | LPC 17:0 | 510.4 | 184.1 | Positive |
| LPC | LPC 17:1 | 508.4 | 184.1 | Positive |
| LPC | LPC 18:0 | 524.4 | 184.1 | Positive |
| LPC | LPC 18:1 | 522.4 | 184.1 | Positive |
| LPC | LPC 18:2 | 520.3 | 184.1 | Positive |
| LPC | LPC 18:3 | 518.3 | 184.1 | Positive |
| LPC | LPC 19:0 | 538.4 | 184.1 | Positive |
| LPC | LPC 19:1 | 536.4 | 184.1 | Positive |
| LPC | LPC 20:0 | 552.4 | 184.1 | Positive |
| LPC | LPC 20:1 | 550.4 | 184.1 | Positive |
| LPC | LPC 20:2 | 548.4 | 184.1 | Positive |
| LPC | LPC 20:3 | 546.4 | 184.1 | Positive |
| LPC | LPC 20:4 | 544.3 | 184.1 | Positive |
| LPC | LPC 20:5 | 542.3 | 184.1 | Positive |
| LPC | LPC 22:0 | 580.4 | 184.1 | Positive |
| LPC | LPC 22:1 | 578.4 | 184.1 | Positive |
| LPC | LPC 22:5 | 570.4 | 184.1 | Positive |
| LPC | LPC 22:6 | 568.3 | 184.1 | Positive |
| LPC | LPC 24:0 | 608.5 | 184.1 | Positive |
| LPC | LPC 24:1 | 606.5 | 184.1 | Positive |
| LPC | LPC 24:2 | 604.5 | 184.1 | Positive |
| LPC | LPC 26:0 | 636.5 | 184.1 | Positive |
| LPE | LPE 14:0 (IS) | 426.3 | 285.3 | Positive |
| LPE | LPE 16:0 | 454.3 | 313.3 | Positive |
| LPE | LPE 17:0 | 468.3 | 327.3 | Positive |
| LPE | LPE 18:0 | 482.3 | 341.3 | Positive |
| LPE | LPE 18:1 | 480.3 | 339.3 | Positive |
| LPE | LPE 18:2 | 478.3 | 337.3 | Positive |
| LPE | LPE 20:1 | 508.3 | 367.3 | Positive |
| LPE | LPE 20:4 | 502.3 | 361.3 | Positive |
| LPE | LPE 22:1 | 536.4 | 395.4 | Positive |
| LPE | LPE 22:5 | 528.3 | 387.3 | Positive |
| LPE | LPE 22:6 | 526.3 | 385.3 | Positive |
| PC | PC 26:0 (IS) | 650.5 | 184.1 | Positive |
| PC | PC 28:0 | 678.5 | 184.1 | Positive |
| PC | PC 28:1 | 676.5 | 184.1 | Positive |
| PC | PC 29:0 | 692.5 | 184.1 | Positive |
| PC | PC 30:0 | 706.5 | 184.1 | Positive |
| PC | PC 31:0 | 720.6 | 184.1 | Positive |
| PC | PC 31:1 | 718.5 | 184.1 | Positive |
| PC | PC 32:0 | 734.6 | 184.1 | Positive |
| PC | PC 32:1 | 732.6 | 184.1 | Positive |
| PC | PC 32:2 | 730.5 | 184.1 | Positive |
| PC | PC 32:3 | 728.5 | 184.1 | Positive |
| PC | PC 33:0 | 748.6 | 184.1 | Positive |
| PC | PC 33:1 | 746.6 | 184.1 | Positive |
| PC | PC 33:2 | 744.6 | 184.1 | Positive |
| PC | PC 34:0 | 762.6 | 184.1 | Positive |
| PC | PC 34:1 | 760.6 | 184.1 | Positive |
| PC | PC 34:2 | 758.6 | 184.1 | Positive |
| PC | PC 34:3 | 756.6 | 184.1 | Positive |
| PC | PC 34:4 | 754.5 | 184.1 | Positive |
| PC | PC 34:5 | 752.5 | 184.1 | Positive |
| PC | PC 35:0 | 776.6 | 184.1 | Positive |
| PC | PC 35:1 | 774.6 | 184.1 | Positive |
| PC | PC 35:2 | 772.6 | 184.1 | Positive |
| PC | PC 35:3 | 770.6 | 184.1 | Positive |
| PC | PC 35:4 | 768.6 | 184.1 | Positive |
| PC | PC 35:5 | 766.5 | 184.1 | Positive |
| PC | PC 36:0 | 790.6 | 184.1 | Positive |
| PC | PC 36:1 | 788.6 | 184.1 | Positive |
| PC | PC 36:2 | 786.6 | 184.1 | Positive |
| PC | PC 36:3 | 784.6 | 184.1 | Positive |
| PC | PC 36:4 | 782.6 | 184.1 | Positive |
| PC | PC 36:5 | 780.6 | 184.1 | Positive |
| PC | PC 36:6 | 778.5 | 184.1 | Positive |
| PC | PC 37:3 | 798.6 | 184.1 | Positive |
| PC | PC 37:4 | 796.6 | 184.1 | Positive |
| PC | PC 37:5 | 794.6 | 184.1 | Positive |
| PC | PC 37:6 | 792.6 | 184.1 | Positive |
| PC | PC 38:2 | 814.6 | 184.1 | Positive |
| PC | PC 38:3 | 812.6 | 184.1 | Positive |
| PC | PC 38:4 | 810.6 | 184.1 | Positive |
| PC | PC 38:5 | 808.6 | 184.1 | Positive |
| PC | PC 38:6 | 806.6 | 184.1 | Positive |
| PC | PC 38:7 | 804.6 | 184.1 | Positive |
| PC | PC 39:5 | 822.6 | 184.1 | Positive |
| PC | PC 39:6 | 820.6 | 184.1 | Positive |
| PC | PC 40:4 | 838.6 | 184.1 | Positive |
| PC | PC 40:5 | 836.6 | 184.1 | Positive |
| PC | PC 40:6 | 834.6 | 184.1 | Positive |
| PC | PC 40:7 | 832.6 | 184.1 | Positive |
| PC | PC 40:8 | 830.6 | 184.1 | Positive |
| PE | PE 28:0 (IS) | 636.5 | 495.5 | Positive |
| PE | PE 32:0 | 692.5 | 551.5 | Positive |
| PE | PE 32:1 | 690.5 | 549.5 | Positive |
| PE | PE 34:0 | 720.6 | 579.5 | Positive |
| PE | PE 34:1 | 718.5 | 577.5 | Positive |
| PE | PE 34:2 | 716.5 | 575.5 | Positive |
| PE | PE 34:3 | 714.5 | 573.5 | Positive |
| PE | PE 35:1 | 732.6 | 591.5 | Positive |
| PE | PE 35:2 | 730.5 | 589.5 | Positive |
| PE | PE 36:0 | 748.6 | 607.6 | Positive |
| PE | PE 36:1 | 746.6 | 605.6 | Positive |
| PE | PE 36:2 | 744.6 | 603.5 | Positive |
| PE | PE 36:3 | 742.5 | 601.5 | Positive |
| PE | PE 36:4 | 740.5 | 599.5 | Positive |
| PE | PE 36:5 | 738.5 | 597.5 | Positive |
| PE | PE 38:1 | 774.6 | 633.6 | Positive |
| PE | PE 38:2 | 772.6 | 631.5 | Positive |
| PE | PE 38:3 | 770.6 | 629.6 | Positive |
| PE | PE 38:4 | 768.6 | 627.5 | Positive |
| PE | PE 38:5 | 766.5 | 625.5 | Positive |
| PE | PE 38:6 | 764.5 | 623.5 | Positive |
| PE | PE 40:2 | 800.6 | 659.6 | Positive |
| PE | PE 40:4 | 796.6 | 655.6 | Positive |
| PE | PE 40:5 | 794.6 | 653.6 | Positive |
| PE | PE 40:6 | 792.6 | 651.5 | Positive |
| PE | PE 40:7 | 790.5 | 649.5 | Positive |
| PI | PI 25:0 (IS) | 730.4 | 453.4 | Positive |
| PI | PI 32:0 | 828.6 | 551.5 | Positive |
| PI | PI 32:1 | 826.5 | 549.5 | Positive |
| PI | PI 34:0 | 856.6 | 579.5 | Positive |
| PI | PI 34:1 | 854.6 | 577.5 | Positive |
| PI | PI 34:2 | 852.6 | 575.5 | Positive |
| PI | PI 36:0 | 884.6 | 607.6 | Positive |
| PI | PI 36:1 | 882.6 | 605.6 | Positive |
| PI | PI 36:2 | 880.6 | 603.5 | Positive |
| PI | PI 36:3 | 878.6 | 601.5 | Positive |
| PI | PI 36:4 | 876.6 | 599.5 | Positive |
| PI | PI 38:2 | 908.6 | 631.6 | Positive |
| PI | PI 38:3 | 906.6 | 629.6 | Positive |
| PI | PI 38:4 | 904.6 | 627.5 | Positive |
| PI | PI 38:5 | 902.6 | 625.5 | Positive |
| PI | PI 38:6 | 900.6 | 623.5 | Positive |
| PI | PI 40:3 | 934.6 | 657.6 | Positive |
| PI | PI 40:4 | 932.6 | 655.6 | Positive |
| PI | PI 40:5 | 930.6 | 653.6 | Positive |
| PI | PI 40:6 | 928.6 | 651.5 | Positive |
| PS | PS 28:0 (IS) | 680.5 | 495.5 | Positive |
| PS | PS 34:0 | 764.5 | 579.5 | Positive |
| PS | PS 36:1 | 790.6 | 605.6 | Positive |
| PS | PS 36:2 | 788.5 | 603.5 | Positive |
| PS | PS 36:3 | 786.5 | 601.5 | Positive |
| PS | PS 38:4 | 812.5 | 627.5 | Positive |
| PS | PS 38:5 | 810.5 | 625.5 | Positive |
| PS | PS 40:5 | 838.6 | 653.6 | Positive |
| PS | PS 40:6 | 836.5 | 651.5 | Positive |
| SM | SM 24:1 (IS) | 563.4 | 184.1 | Positive |
| SM | SM 30:1 | 647.5 | 184.1 | Positive |
| SM | SM 31:1 | 661.5 | 184.1 | Positive |
| SM | SM 32:0 | 677.6 | 184.1 | Positive |
| SM | SM 32:1 | 675.5 | 184.1 | Positive |
| SM | SM 32:2 | 673.5 | 184.1 | Positive |
| SM | SM 33:1 | 689.6 | 184.1 | Positive |
| SM | SM 33:2 | 687.6 | 184.1 | Positive |
| SM | SM 34:0 | 705.6 | 184.1 | Positive |
| SM | SM 34:1 | 703.6 | 184.1 | Positive |
| SM | SM 34:2 | 701.6 | 184.1 | Positive |
| SM | SM 34:3 | 699.5 | 184.1 | Positive |
| SM | SM 35:1 | 717.6 | 184.1 | Positive |
| SM | SM 35:2 | 715.6 | 184.1 | Positive |
| SM | SM 36:0 | 733.6 | 184.1 | Positive |
| SM | SM 36:1 | 731.6 | 184.1 | Positive |
| SM | SM 36:2 | 729.6 | 184.1 | Positive |
| SM | SM 36:3 | 727.6 | 184.1 | Positive |
| SM | SM 37:1 | 745.6 | 184.1 | Positive |
| SM | SM 37:2 | 743.6 | 184.1 | Positive |
| SM | SM 38:0 | 761.6 | 184.1 | Positive |
| SM | SM 38:1 | 759.6 | 184.1 | Positive |
| SM | SM 38:2 | 757.6 | 184.1 | Positive |
| SM | SM 38:3 | 755.6 | 184.1 | Positive |
| SM | SM 39:1 | 773.7 | 184.1 | Positive |
| SM | SM 39:2 | 771.7 | 184.1 | Positive |
| SM | SM 40:0 | 789.7 | 184.1 | Positive |
| SM | SM 40:1 | 787.7 | 184.1 | Positive |
| SM | SM 40:2 | 785.7 | 184.1 | Positive |
| SM | SM 40:3 | 783.7 | 184.1 | Positive |
| SM | SM 41:1 | 801.7 | 184.1 | Positive |
| SM | SM 41:2 | 799.7 | 184.1 | Positive |
| SM | SM 42:0 | 817.7 | 184.1 | Positive |
| SM | SM 42:1 | 815.7 | 184.1 | Positive |
| SM | SM 42:2 | 813.7 | 184.1 | Positive |
| SM | SM 42:3 | 811.7 | 184.1 | Positive |
| SM | SM 43:1 | 829.7 | 184.1 | Positive |
| SM | SM 43:2 | 827.7 | 184.1 | Positive |
| SM | SM 44:1 | 843.7 | 184.1 | Positive |
| SM | SM 44:2 | 841.7 | 184.1 | Positive |
| SM | SM 44:3 | 839.7 | 184.1 | Positive |

References

1.	Wei R, Wang J, Su M, Jia E, Chen S, Chen T, Ni Y. Missing Value Imputation Approach for Mass Spectrometry-based Metabolomics Data. Sci Rep. 2018;8(1):663.

2.	Dieterle F, Ross A, Schlotterbeck G, Senn H. Probabilistic quotient normalization as robust method to account for dilution of complex biological mixtures. Application in 1H NMR metabonomics. Anal Chem. 2006;78(13):4281-90.

3.	World Health Organization. Global Recommendations on Physical Activity for Health. Geneva: World Health Organization; 2010.

4.	Rasmussen KM, Yaktine AL, editors. Weight Gain During Pregnancy: Reexamining the Guidelines. Washington (DC): National Academies Press (US); 2009.

5.	Mikolajczyk RT, Zhang J, Betran AP, Souza JP, Mori R, Gülmezoglu AM, Merialdi M. A global reference for fetal-weight and birthweight percentiles. Lancet. 2011;377(9780):1855-61.

6.	Pang WW, Aris IM, Fok D, Soh SE, Chua MC, Lim SB, et al. Determinants of Breastfeeding Practices and Success in a Multi-Ethnic Asian Population. Birth. 2016;43(1):68-77.
